# *HERC5* is a prognostic biomarker for post-liver transplant recurrent human hepatocellular carcinoma

**DOI:** 10.1186/s12967-015-0743-2

**Published:** 2015-12-11

**Authors:** Feng Xue, Brandon W. Higgs, Jiaqi Huang, Chris Morehouse, Wei Zhu, Xin Yao, Philip Brohawn, Zhan Xiao, Yinong Sebastian, Zheng Liu, Yun Xia, Dong Shen, Mike Kuziora, Zhengwei Dong, Hulin Han, Yi Gu, Jianren Gu, Qiang Xia, Yihong Yao

**Affiliations:** Department of Liver Surgery and Liver Transplantation, Renji Hospital, Shanghai Jiaotong University School of Medicine, Shanghai, 201203 China; Translational Bioinformatics, MedImmune, Gaithersburg, MD 20878 USA; Asia and Emerging Markets iMed, AstraZeneca R&D, 199 Liangjing Road, Shanghai, 201203 China; Shanghai Cancer Institute, Renji Hospital, Shanghai Jiaotong University School of Medicine, Shanghai, 201203 China

**Keywords:** Hepatocellular carcinoma, Orthotopic liver transplantation, Whole exome, *HERC5*

## Abstract

**Background and aims:**

Orthotopic liver transplantation (OLT) can be an effective treatment option for certain patients with early stage hepatocellular carcinoma (HCC) meeting Milan, UCSF, or Hangzhou criteria. However, HCC recurrence rates post-OLT range from 20 to 40 %, with limited follow-up options. Elucidating genetic drivers common to primary and post-OLT recurrent tumors may further our understanding and help identify predictive biomarkers of recurrence—both to ultimately help manage clinical decisions for patients undergoing OLT.

**Methods:**

Whole exome and RNA sequencing in matched primary and recurrent tumors, normal adjacent tissues, and blood from four Chinese HCC patients was conducted. SiRNA knockdown and both qRT-PCR and Western assays were performed on PLCPRF5, SNU449 and HEPG2 cell lines; immunohistochemistry and RNA Sequencing were conducted on the primary tumors of Chinese HCC patients who experienced tumor recurrence post-OLT (n = 9) or did not experience tumor recurrence (n = 12).

**Results:**

In three independent HCC studies of patients undergoing transplantation (n = 21) or surgical resection (n = 242, n = 44) of primary tumors (total n = 307), *HERC5* mRNA under-expression correlated with shorter: time to tumor recurrence (p = 0.007 and 0.02) and overall survival (p = 0.0063 and 0.023), even after adjustment for relevant clinical variables. *HERC5* loss drives CCL20 mRNA and protein over-expression and associates with regulatory T cell infiltration as measured by FOXP3 expression. Further, matched primary and recurrent tumors from the 4 HCC patients indicated clonal selection advantage of Wnt signaling activation and CDKN2A inactivation.

**Conclusions:**

HERC5 plays a crucial role in HCC immune evasion and has clinical relevance as a reproducible prognostic marker for risk of tumor recurrence and survival in patients.

**Electronic supplementary material:**

The online version of this article (doi:10.1186/s12967-015-0743-2) contains supplementary material, which is available to authorized users.

## Background

Hepatocellular carcinoma (HCC) is the most common primary liver malignancy and third leading cause of cancer deaths worldwide, with Hepatitis B virus a major etiological factor [1, 2]. Beyond sorafenib (Bayer HealthCare Pharmaceuticals, Inc.; Onyx Pharmaceuticals, Inc, Germany) which is only effective in a small patient population, there is no approved treatment for HCC. Patients have limited options, and orthotopic liver transplantation (OLT) is viable for certain early stage HCC cases, though it is only efficacious in a subset meeting Milan, Toronto, or UCSF clinical criteria [3–5]. The HCC recurrence rates after OLT range from 20 to 40 %, and treatment options after recurrence are limited [3–5]. To date, certain clinicopathologic variables such as tumor size and absence of macroscopic vascular invasion are used to predict risk of recurrence, though success of these factors vary from study-to-study [6]. In an effort to improve the prediction of HCC recurrence, molecular profiling has been applied in many studies.

Multiple transcriptomic and proteomic studies have been conducted to help understand the link between molecular mediators and factors of etiology, tumorigenesis, disease course, and/or other variables related to survival and recurrence in HCC. In fact, from 2003 to 2010, over 14 studies have identified gene signatures from a minimum of 12 genes or proteins to 186, totally more than 934 genes for purposes of predicting survival and/or recurrence in HCC patients [7–21]. More recently, Kim et al., developed a 233 gene signature to discern early from late tumor recurrence in primarily HBV-positive HCC [22], while Zheng et al., combined a 122 gene hepatic stellate cell signature with clinical variables for a prognostic index to predict overall survival in HCV-positive cirrhosis or HCC patients [23]. In contrast to gene or protein signatures, single analyte prognostic gene expression markers such as TNF-related apoptosis-inducing ligand (*TRAIL*) mRNA was shown to associate with tumor growth and survival, though the latter result did not show statistical significance, and melanoma-associated antigen-D2 (*MAGE*-*D2*) mRNA was identified by Hashimoto et al., as a prognostic factor for disease-specific survival following curative hepatectomy [24, 25] The commonality of genes across these studies is low, primarily due to factors of: heterogeneity within HCC populations, degraded RNA isolated from formalin-fixed tissues, differences in clinical stages and etiologies, small sample sizes, lack of independent validation, and basic analytical strategy used to identify predictive genes. Beyond gene or protein expression patterns shown in these studies, the underlying genetic role in HCC recurrence and how it influences pathway modulation has not been explored—something that can greatly enhance our ability to accurately predict tumor recurrence in HCC.

Recent sequencing studies have advanced our knowledge of genetic oncodrivers in HCC, identifying the most recurrent functional impacting mutations in genes and frequently modulated pathways such as Wnt signaling, G1/S cell cycle signaling, apoptosis, and JAK/STAT signaling [26–29]. Additional work has helped elucidate both sites and functional effects of the viral-host genome integration for HBV within HCC patients [30, 31]. These studies have provided a foundation for the genetic landscape of primary tumors in HCC patients though the genetic basis leading to tumor recurrence remains poorly understood, particularly somatic variation shared between the primary and recurring tumors and mechanisms supporting certain predictors of survival or disease recurrence, beyond statistical correlates.

In this study, we used an integrated omics strategy to identify a hemizygous DNA deletion and concordant mRNA under-expression of *HERC5*, an IFN-induced HECT-type E3 protein ligase gene associated with shorter: time to tumor recurrence and overall survival in HCC patients. The downstream immune-pathological impact from loss of *HERC5* was also determined. Additionally, this study indicates a clonal selection advantage in the genetic changes in Wnt signaling in the recurrent tumors, relative to primary tumors of HCC patients. Assessing risk of such outcomes in HCC is a significant unmet need and a predictive biomarker to help manage clinical decisions has high relevance for patients potentially undergoing OLT.

## Patients and methods

### Patients and samples

To adhere to REporting recommendations for tumour MARKer prognostic studies (REMARK) reporting of clinical specimens, 21 patients within the Hangzhou criteria [4] who underwent OLT at Renji Hospital from 2008 to 2012 were retrospectively included in this study. No stratification or matching was used for patient inclusion in this study. Informed written consent was obtained from each patient and the study protocol conformed to the ethical guidelines of the 1975 Declaration of Helsinki as reflected in a priori approval by the Ethics Committee of Renji Hospital. No donor organs were obtained from executed prisoners or other institutionalized persons. Within 24 months after OLT, 9 patients had recurrent liver tumors or remote metastasis following OLT, while the remaining 12 patients were tumor free. All clinical variables considered are provided in Table 1 and further detailed in Additional file 1: Table S1. The primary tumor (P_T_) and normal adjacent tissue (P_NAT_) were collected from all patients (n = 21). The recurrent tumor (R_T_), normal adjacent tissue from donor (R_NAT_), and recipient blood (P_B_) were collected from 4 of 9 recurrent patients.

**Table 1:** The summary of clinical information for 21 Chinese HCC patients

Patient	Recurrent with 24 months±	Sex	Age	Primary tumor grade	Child	BCLC	Primary tumor size (cm)	Criteria (Milan = 1; UCSF = 2; Exceed = 3)	AFP (ng/mL)	MELD	Time of recurrent after OLT (month)	Recurrent tumor organ	HBV (pre-OLT)	HBV (post-OLT)	MULT±	SAT±	ENCAP±	CIRR±	VES±	THROMB±
HCC1*	1	M	48	III	A	A3	5*5*3	1	2935.9	8	8	Liver	Positive	Negative	0	0	0	1	0	1
HCC4*	1	M	59	II	A	B	8*6*5.5	3	2.5	5	6.5	Liver	Positive	Positive	1	1	1	0	0	0
HCC5*	1	M	67	II	A	B	5*4*3	1	>3000	2	18	Liver	Negative	Negative	1	0	0	1	0	1
HCC11*	1	F	57	II–III	A	B	0.5–2.5	1	1581.6	2	16	Lung	Negative	Negative	1	0	0	1	0	0
HCC2	1	M	43	II	A	B	9*8*6	3	1461.1	21	19	Liver	Positive	Negative	0	1	N/A	1	0	0
HCC3	1	M	42	III	B	B	6*6*2.5, 10*8*4	3	>3000	5	14.5	Liver	Positive	Negative	1	0	0	1	0	1
HCC6	1	M	41	III	A	B	7*5*4	3	126	4	1.5	Lung	Positive	Negative	0	0	0	1	0	0
HCC8	1	M	42	III	A	B	10*10*7	3	>3000	5	11	Liver	Positive	Negative	0	1	0	1	N/A	1
HCC10	1	M	59	III	A	A3	4*3.5*3.5	1	1888.2	15	6	Liver	Positive	Negative	0	0	0	1	0	1
*HCC-C2*	*0*	*M*	*55*	*I–II*	*C*	*D*	5.5*5*4.5*	*2*	*5.2*	*4*	*N/A*	*N/A*	*Positive*	*Negative*	*0*	*0*	*0*	*1*	*0*	*0*
*HCC-C6*	*0*	*M*	*51*	*II*	*B*	*A3*	3*2.5*2*	*1*	*137.5*	*13*	*N/A*	*N/A*	*Positive*	*Negative*	*0*	*0*	*0*	*1*	*0*	*0*
*HCC-C8*	*0*	*M*	*64*	*I*	*B*	*A3*	5*1.5*1.5*, 3*2*2*	*3*	*62.5*	*13*	*N/A*	*N/A*	*Positive*	*Positive*	*1*	*0*	*0*	*1*	*0*	*0*
*HCC-C9*	*0*	*M*	*40*	*II*	*B*	*A3*	1*1*1*	*1*	*9.2*	*3*	*N/A*	*N/A*	*Positive*	*N/A*	*0*	*0*	*0*	*1*	*0*	*0*
*HCC-C11*	*0*	*M*	*62*	*I*	*A*	*A3*	3*3*2*	*1*	*5.4*	*9*	*N/A*	*N/A*	*Positive*	*Negative*	*0*	*0*	*0*	*1*	*0*	*0*
*HCC-C12*	*0*	*M*	*40*	*II–III*	*A*	*A2*	2.5*1.5*1*	*1*	*210.8*	*4*	*N/A*	*N/A*	*Positive*	*Positive*	*0*	*0*	*0*	*1*	*0*	*0*
*HCC-C13*	*0*	*M*	*49*	*I–II*	*A*	*C*	3*2.5*2.5*; 2*2*2*	*1*	*4.3*	*4*	*N/A*	*N/A*	*Positive*	*Negative*	*1*	*0*	*0*	*1*	*1*	*0*
*HCC-C14*	*0*	*M*	*59*	*II*	*B*	*B*	7*5*5*	*3*	*240.7*	*20*	*N/A*	*N/A*	*Positive*	*N/A*	*0*	*0*	*0*	*1*	*0*	*0*
*HCC-C15*	*0*	*M*	*49*	*II*	*A*	*B*	5*4*2*; 5*4.5*3*	*3*	*NA*	*4*	*N/A*	*N/A*	*Positive*	*Negative*	*0*	*1*	*0*	*1*	*0*	*0*
*HCC-C16*	*0*	*M*	*49*	*II–III*	*B*	*A3*	5*5*4*	*1*	*128.9*	*11*	*N/A*	*N/A*	*Positive*	*N/A*	*0*	*1*	*0*	*1*	*0*	*0*
*HCC-C18*	*0*	*M*	*57*	*I*	*A*	*A2*	5*4*4*	*1*	*6.6*	*2*	*N/A*	*N/A*	*Positive*	*Negative*	*0*	*0*	*0*	*1*	*0*	*0*
*HCC-C19*	*0*	*M*	*41*	*II–III*	*A*	*B*	4*2.5*2*, 3*2*1.8*	*2*	*220.9*	*2*	*N/A*	*N/A*	*Positive*	*Positive*	*1*	*0*	*0*	*1*	*0*	*0*

Patient OLT inclusion criteria included: ECOG (Eastern Cooperative Oncology Group performance status) score 0–2, tumor within Hangzhou criteria and no major vascular invasion or extra hepatic metastases from imaging studies. Exclusion criteria included absolute contradictions of: involvement of the surrounding tissue or distant metastasis, co-current non-curable extra hepatic malignancies, active infection; relative contraindications included: pulmonary hypertension, symptomatic ischemic heart disease, severe renal insufficiency, and mental disorders

*AFP* alpha-fetoprotein, *HBV* hepatitis B virus, *MULT* multiplicity, *ENCAP* encapsulated, *CIRR* cirrhosis, *THROMB* thrombosis, *OLT* orthotopic liver transplantation, *BCLC* barcelona Clinical Liver Cancer staging, *SAT* satellite, *VES* vessel, *Child* Child-Pugh score, *MELD* Model for End-Stage Liver Disease, *±1* Yes, *0* No

* These 4 patients have matched primary and recurrent tumor specimens, normal adjacent tissue from the primary and recurrent tumors, and recipient blood specimens (n = 20 total specimens)

Italicized rows indicate patients who did not experience tumor recurrence post-OLT (n = 12), while non-italicized rows indicate patients who did experience tumor recurrence post-OLT (n = 9)

### DNA sequence, read mapping and variant calling

DNA exome sequencing (WES) was generated by Beijing Genomics Institute (BGI) using the Illumina standard library preparation and sequencing protocols [30]. Paired-end 90mer sequence FASTQs for both data types were provided to MedImmune. WES data was available from four patients, all of whom experienced tumor recurrence post-OLT with the following specimens: P_T_, R_T_, P_NAT_, R_NAT_, and P_B_. QC and both patient-level and summarized variants results are provided in Additional file 1: Tables S2–S5. Detailed explanations of somatic and germline variant and indel calling is provided in Additional file 2, in addition to the following methods: Patient identity, Clonal relationship value derivation, Donor tissue presence in recurrent tumors, Somatic copy number variation (CNV) analysis, Specificity of HERC5 prognostic correlation among genes in chr4q, and Integrated pathway analysis (Additional file 1: Tables S6–S8).

### RNASeq read mapping and differential expression analysis

RNASeq data was generated by BGI using the Illumina standard library preparation and sequencing protocols [30]. Paired end 90mer sequence FASTQs were provided to MedImmune. Sequence data was QCd using FastQC (v0.10.1), with average read count per mate 50 million. Reads were mapped to reference (UCSC hg19; Feb 2009 release; Genome Reference Consortium GRCh37) using TopHat2 (v2.0.9; 32) using the human reference gtf annotation file (GRCh37.68). Transcript counts were calculated/normalized using htseq-count and DESeq (v1.12.1; 33). DESeq’s negative binomial distribution was used to calculate p-values and fold changes between P_T_ and P_NAT_ as well as R_T_ and R_NAT_ using p < 0.01 and |fold| >2 as a threshold for the four patients. These results were used in the pathway analyses and combined with the same genes harboring copy number (CN) amplified or deleted regions (see Additional file 2 for CN calling methods). Unadjusted p-values were utilized to simply identify the most differential transcripts within a single patient (P_T_ vs. P_NAT_) using the fold change magnitude as a primary gene ranking. Since *p* value calculations were conducted within each patient, there was no replication and statistical power was not adequate to warrant multiple testing adjustment. Tumor cell prevalence was evaluated using ABSOLUTE [34] and verified against pathology assessments for each tumor. RNASeq data was available from 21 patients (9 experienced tumor recurrence post-OLT and 12 did not) with the following specimens: P_T_ and P_NAT_.

### Time-to-event analyses

Time-to-event analyses were used to correlate the expression of the four genes (*NAA11*, *HERC5*, *DDX60*, and *HERC6)* identified with tumor recurrence among the 21 Chinese patients’ primary tumors from this study (n = 9 experienced tumor recurrence; n = 12 did not experience tumor recurrence). The expression of each gene in the normal adjacent tissue was subtracted from the tumor expression for each patient (P_T_-P_NAT_) individually, then each gene was cut at the median into high or low expression groups. In alignment with REMARK criteria, Kaplan–Meier (KM) analysis, univariate Cox proportional hazards (PH) regression, and multivariate Cox PH regression analyses were conducted adjusting for HBV status post-OLT, age (binary), gender, and tumor grade. These four variables were the most relevant for potential confounding factors with a molecular prognostic. Cirrhosis status was positive for all but one patient, so this variable was not used in the analysis of these 21 patients. *HERC5* was the only significant correlate (p < 0.05) with tumor recurrence among the four genes using a KM model (Fig. 3a; Table 2).

**Table 2:** Univariate Kaplan–Meier (KM) and Cox proportional hazards (PH) models and multivariate Cox PH models for the 21 Chinese HCC patients in this study, HCC patients from Roessler et al. study, and HCC patients from Boyault et al. study

21 Chinese patients	Recurrence (n = 21; 9 events)	
	HR (95 % CI)	p value
Univariate Cox PH		
HBV pre-OLT (Yes, No)	0.285 (0.06, 1.48)	0.14
Tumor grade (GI, GII GIII)	1.27 (0.63, 2.01)	4.50E−02
Age (<49)	2.33 (0.62, 8.68)	0.21
Gender (M, F)	0.29 (0.03, 2.47)	0.26
*HERC5* (Low, High)	10.34 (1.28, 83.55)	0.029
Univariate KM		
*HERC5* (Low, High)		0.007
Multivariate Cox PH		
*HERC5* (Low)	7.29	0.07
HBV pre-OLT (Yes, No)	0.26	0.3
Tumor grade	2.41	0.27
Age (<49)	2.08	0.42
Gender (M, F)	1.86	0.7

Roessler et al. study	Survival (n = 224; 86 events)		Recurrence (n = 224; 125 events)	
	HR (95 % CI)	p value	HR (95 % CI)	p value
Univariate Cox PH				
HBV (AVR-CC, CC, No)	1.32 (0.86, 2.03)	0.212	1.24 (0.86, 1.79)	0.26
TNM Staging (I, II, III)	2.34 (1.77, 3.09)	2.18E−09	1.76 (1.41, 2.20)	7.81E−07
Age (<50)	1.26 (0.84, 1.88)	0.262	1.01 (0.72, 1.42)	0.96
Gender (M, F)	1.86 (0.09, 3.83)	0.0933	2.36 (1.24, 4.50)	0.009
Cirrhosis (No, Yes)	0.20 (0.05, 0.80)	0.0227	0.50 (0.23, 1.07)	0.07
*HERC5* (Low, High)	1.79 (1.17, 2.74)	0.00706	1.55 (1.07, 2.24)	0.021
Univariate KM				
*HERC5* (Low, High)		0.0063		0.0198
Multivariate Cox PH				
*HERC5* (Low)	2.02	0.004	1.8	0.004
Gender (M, F)	1.36	0.42	2.07	0.03
Cirrhosis (No, Yes)	0.28	0.076	0.56	0.17
Age (<50)	1	0.99	1.01	0.49
HBV (AVR-CC, CC, No)	1.3	0.25	1.36	0.11
TNM Staging (I, II, III)	2.32	1.06E−08	1.74	2.21E−06

Boyault et al. study	Survival (n = 41; 20 events)		PFS (n = 41; 20 events)	
	HR (95 % CI)	p value	HR (95 % CI)	p value
Univariate Cox PH				
HBV (Yes, No)	1.32 (0.51, 3.40)	0.57	1.02 (0.44, 2.38)	0.96
Gender (M, F)	1.01 (0.34, 3.03)	9.88E−01	1.30 (0.49, 3.41)	6.01E−01
Age (<65)	1.10 (0.45, 2.67)	0.85	1.48 (0.70, 3.17)	0.31
*HERC5* (Low, High)	2.69 (1.11, 6.51)	0.029	1.96 (0.95, 4.05)	0.07
Univariate KM				
*HERC5* (Low, High)		0.023		0.07
Multivariate Cox PH				
*HERC5* (Low)	3.31	0.018	3.8	0.01
HBV (Yes, No)	1.28	0.63	1.16	0.78
Gender (M, F)	1.22	0.74	1.32	0.64
Age (<65)	1.02	0.42	1.02	0.4

Models indicate predictions of survival, PFS, or HCC recurrence with *HERC5* mRNA expression and other relevant clinical factors

Tumor grade is defined by American Joint Committee on Cancer. *AJCC Cancer Staging Manual*. 7th ed. New York, NY: Springer; 2010; TNM staging levels are defined by the TNM combinations corresponding to one of five stages (stages I–V)

*AVR-CC* active viral replication chronic carrier, *CC* chronic carrier, *No* no HBV

Then, correlation between under-expression of *HERC5* and both progression-free survival (PFS) and overall survival was conducted in a publically available HCC microarray dataset [35, E-TABM-36]. This *HERC5* under-expression was also tested again with both HCC recurrence and overall survival in an additional publically available microarray dataset [36, GSE14520]. As was conducted for the 21 HCC patients in this study, the three different analysis models (KM model, univariate Cox PH regression, and multivariate Cox PH regression) were used to test association of *HERC5* under-expression with outcomes in these additional studies (Table 2). For study [36], liver tissue from healthy donors was available, so *HERC5* (219863_at) was divided into high or low patient groups using mean-2 standard deviations (SD) of the normal liver distribution (n = 239) as the cut point (n = 62 and 180 HCC patients in low or high groups, respectively [18 patients were missing clinical data]). The difference between these groups was assessed using the grouping coefficient p-value, hazard ratio, and likelihood ratio test, in multivariate Cox PH regression with the available variables of age, cirrhosis (binary), gender (binary), HBV/HCV status (active viral replication chronic carrier = 2; chronic carrier = 1; no = 0), and TNM staging (I, II, or III). The same variables were also assessed individually in univariate Cox PH regression. Both overall survival and time to tumor recurrence were assessed in two separate analyses (Fig. 3b; Table 2). For study [35], no normal healthy tissue (matched nor independent subject as in study [36]) was available, so *HERC5* was cut into high or low groups using the median of the HCC primary tumor expression values (n = 20 and 24 HCC patients in low or high groups, respectively [four patients were missing clinical data]). Then overall survival and PFS between high and low patient groups was computed adjusting for the available variables of gender (binary), age, and HBV (titer negative = 0; titer positive = 1) status (Fig. 3c; Table 2). Note that all available clinical variables in both microarray validation studies were analyzed with both univariate and multivariate Cox PH regressions with *HERC5* to assess contribution of these variables to prognostic outcomes. All model summaries are reported in Table 2.

### Biological significance of HERC5 loss in HCC

HERC5 siRNA transfection experiments, Microarray study of HERC5 siRNA knockdown in HCC cell lines, qRT-PCR (TaqMan) Validation, and Cell culture, ELISA assays and FOXP3 IHC assay on P_T_ and P_NAT_ samples are described in Additional file 2.

## Results

### Identification of tumor origin and estimation of donor cell contamination in the recurrent tumors of HCC patients post-OLT

Cases of donor-transmitted malignancies in cadaveric organ transplants are very rare [37, 38] in that recurrent HCC cases post-OLT are likely tumors derived from the recipient, thus the recurrent tumor (R_T_) genetic composition should match the primary tumor (P_T_). Previous studies have used microsatellite markers or CNVs to answer this question of tumor origin post-OLT or resection in HCC, as it has important implications for clinical and therapeutic strategies [37–39]. We implemented a derivation of the clonal relationship value [CR, 39] to determine the tumor origin in the R_T_s for the four patients—all four were of recipient origin (Additional file 2, Additional file 3: Figure S5).

Single nucleotide variants (SNVs) were used to assess donor tissue contamination within each R_T_. Since all four R_T_s were determined to originate from the recipient, any clonality difference between the P_T_s and R_T_s, such as clonal frequencies is due to purity of the R_T_ biopsy [40]. Donor cell contamination in the capture of the R_T_ can dilute the magnitude of somatic differences. Elimination of recipient versus donor-identified differences [normal adjacent tissue from donor (R_NAT_) vs. recipient blood (P_B_)] controls for some of this contamination, though the distribution of cancer clones will still vary between the P_T_s and R_T_s. The malignancy in patient HCC11 occurred in the lung, thus, both primary and metastatic tumors were of recipient origin, serving as a negative control against the other three patients. Using two independent approaches—somatic SNVs or germline SNPs, for each patient (Additional file 2), we estimated the proportion of donor cell contamination in the R_T_s as: HCC1 = 72–86 %; HCC4 = 3–9 %; HCC5 = 48–64 %; and HCC11 = 0 % (recipient = donor; Additional file 3: Figure S6A–B).

### Somatic single nucleotide variant and insertion/deletion identification in primary and recurrent tumors of 4 HCC patients

We first asked whether there were potential major drivers shared between the P_T_s and R_T_s in HCC. Following quality control of the WES and RNASeq data (Additional file 3: Figures S2, S3), we identified 1,145 somatic variants using a stringent selection approach and controlling for donor-recipient differences (i.e. elimination of R_NAT_ vs. P_B_ variants; Additional file 2) in both P_T_s and R_T_s including 616 somatic nonsilent (nonsynonymous, stop-gain, stop-loss, or frameshift substitution) SNVs or insertion/deletions (indels) affecting 567 genes (Additional file 1: Tables S3–S5). On average 123 and 121 nonsilent somatic SNVs and 5 and 4.3 indels were identified, respectively in the P_T_s and R_T_s—an average of 96 SNVs and 3.5 indels shared between these tumors (Additional file 1: Table S3). The somatic SNV distribution in P_T_ and R_T_ specimens had highest occurrences of C > T/G > A and lowest occurrences of T > G/A > C (Additional file 3: Figure S4), consistent with a previous HCC report of coding exons [27], and P_T_ and R_T_ pairs for each patient had similar distributions of transition/transversion substitutions, though it is interesting that HCC11 showed highest prevalence of T > A/A > T. The nonsilent-to-silent SNV rate was average of 2.8 in the P_T_s and 2.3 in the R_T_s—a lower ratio in the R_T_s due to donor-tissue contamination ( Additional file 1: Table S3, Additional file 2). Nonsilent variants shared between P_T_s and R_T_s had a higher proportion of clones compared to each unique set (Additional file 3: Figure S1A-D) and variant allele frequencies (Vfs) for these shared SNVs were significantly higher (mean = 39.4) than those unique to P_T_s (mean = 23.4; p < 0.001 all four patients; Additional file 1: Table S3; Additional file 3: Figure S1E), suggesting shared somatic mutations to likely be driver mutations. The SNVs in the P_T_s called using WES were confirmed at certain loci with adequate depth and quality using WGS and RNASeq data (Additional file 1: Table S4).

### Somatic copy number variant (CNV) detection in the primary, recurrent, and shared tumors of HCC patients

**Fig. 1:**
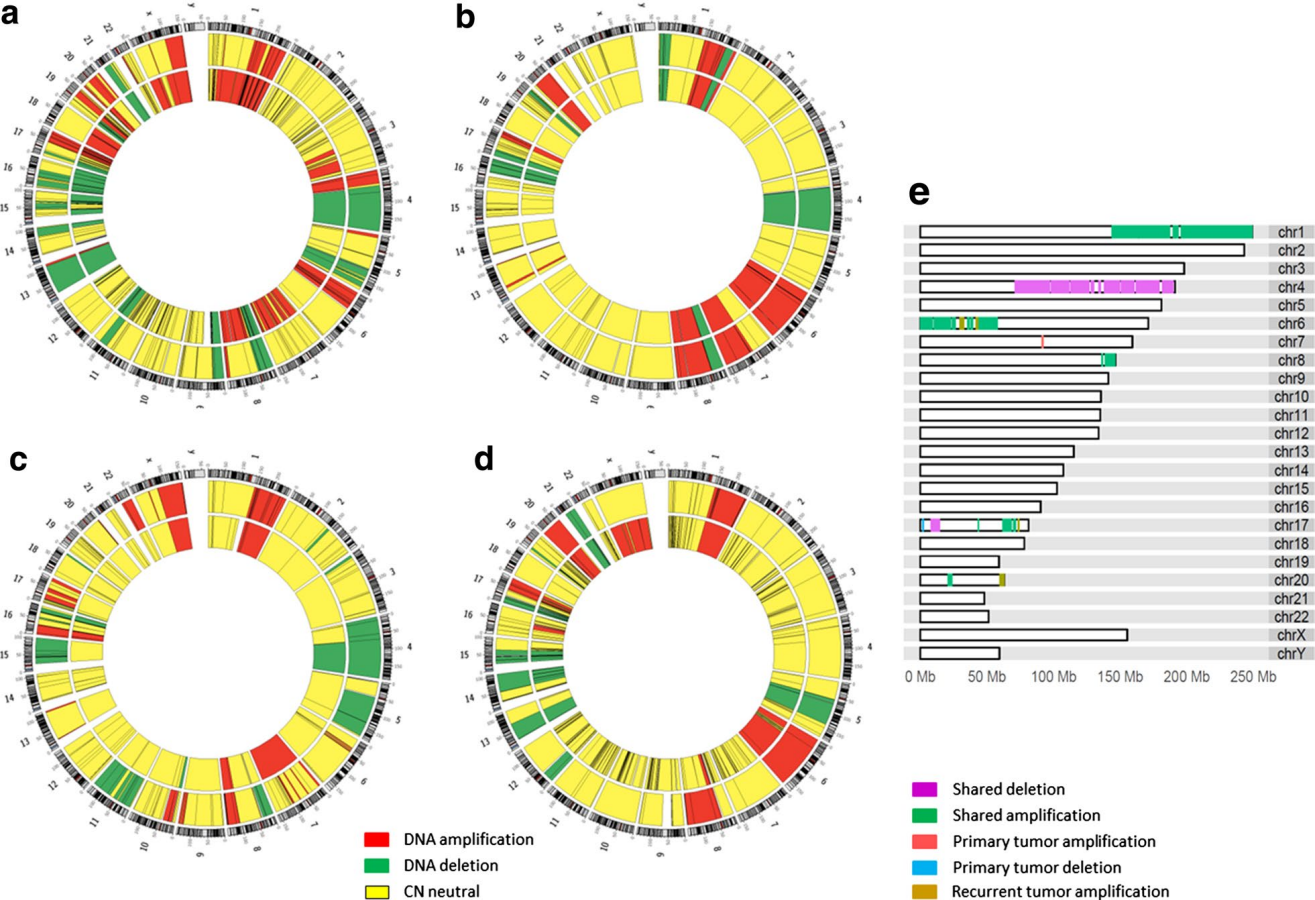
Somatic copy number amplifications (*red*), deletions (*green*), or neutral (*yellow*) identified in the primary (inner track) and recurrent (outer track) tumors of **a** HCC1, **b** HCC4, **c** HCC5, and **d** HCC11; **e** both shared and unique regions of CN amplifications or deletions in 3/4 patients across the genome. *Color code* is as follows: unique to primary tumors (*red* amplification; *blue* deletion); unique to recurrent tumors (*brown* amplification); and shared by the primary and recurrent tumors (*green* amplification; *purple* deletion)

Somatic copy number (CN) amplifications or deletions were selected for uniqueness to the P_T_, R_T_, or shared between the two, all within at least 3 of 4 patients (Fig. 1; “Patients and methods”). Among the hemizygous (hemi) or homozygous (homo) amplifications identified, those that have been observed in a previous study of primary tumors in HCC using comparative genomic hybridization [39] are indicated by an asterisk (*). Those amplifications that were common to both the P_T_ and R_T_ in this study included: 1q* (hemi in all patients), 6p* (homo in 2 patients; hemi in 1 patient), 8q* (homo in 1 patient; hemi in 2 patients), 17q* (hemi in 3 patients), and 20p* (hemi in 3 patients). Common hemi or homo deletions identified in P_T_ and R_T_ included: 4q* (hemi in 3 patients) and 17p* (hemi in 3 patients), and amplified regions unique to R_T_s included 17q (hemi in all patients) and 20q (hemi in all patients). Thus, the vast majority of somatic CN amplifications or deletions were shared between the P_T_ and R_T_, with two short regions having unique CN amplifications in the R_T_.

### An integrated genetics and genomics analysis identifies Wnt-signaling pathway activation and tumor suppressor gene CDKN2A inactivation shared in both P_T_s and R_T_s of HCC patients

**Fig. 2:**
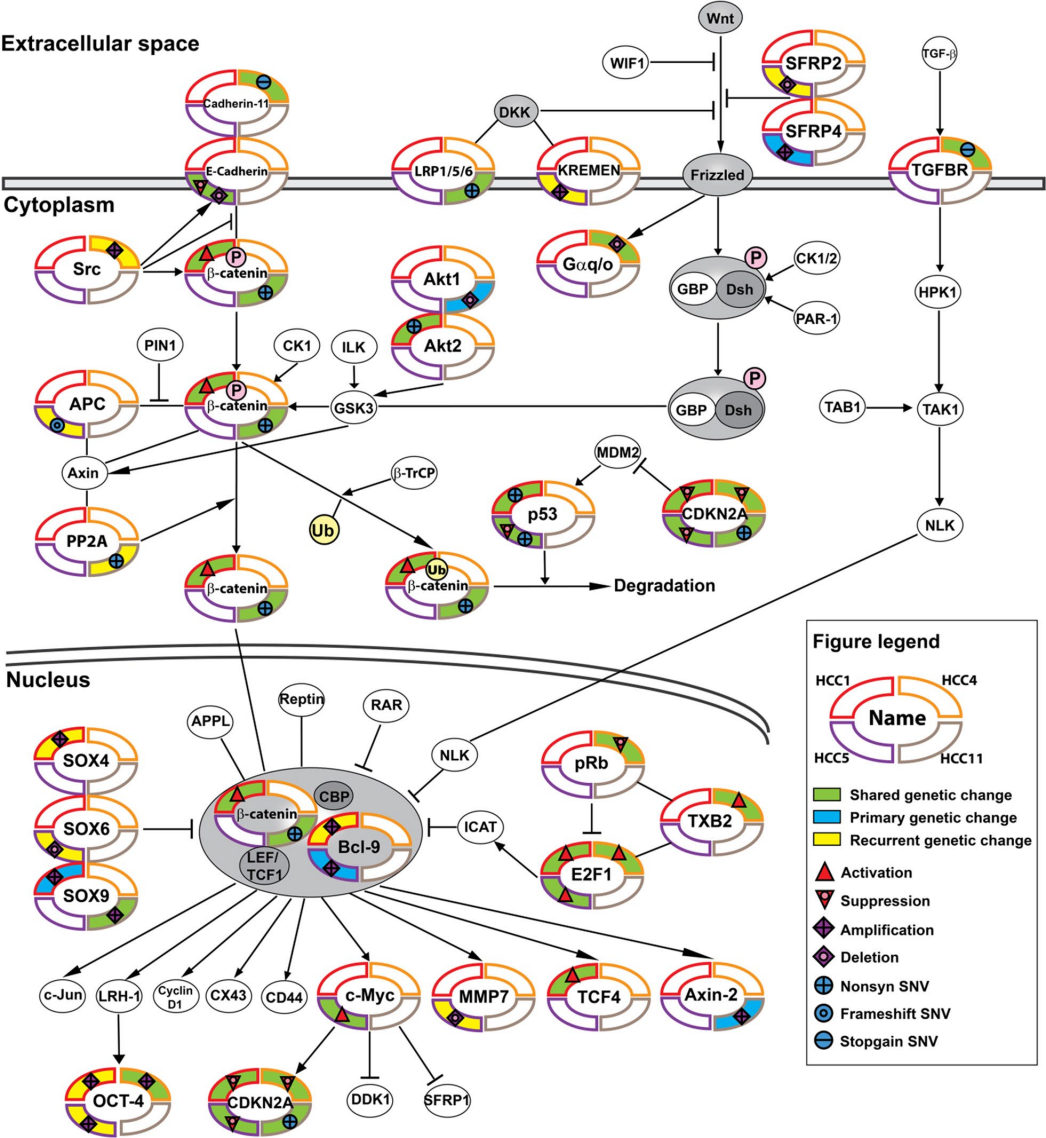
Wnt/β-catenin signaling and directly related pathways affected by genetic-driven and/or gene expression activation or suppression in primary and/or recurrent tumors of 4 HCC patients. Determination of this pathway as *most activated* using genetic and genomic data is described in “Patients and methods” while other top ranked pathways are presented in Additional file 1: Table S6. Each of the 4 patients’ primary or recurrent tumors are represented as a quadrant on each pathway node

Using somatic variants, CNVs, and gene expression from P_T_s and R_T_s (Additional file 3: Figure S7; “Patients and methods”), Wnt/β-catenin signaling was identified as the most altered pathway in both P_T_s and R_T_s across all four patients (Additional file 1: Table S6). Although few affected genes were shared between patients, all identified genetic changes had the potential to prevent ubiquitin-mediated degradation of beta-catenin in the cytoplasm, drive activation of beta-catenin in the nucleus (Fig. 2), and subsequent loss of cell-cycle control by inactivation of CDKN2A.

Specifically, deleterious somatic genetic or genomic alterations shared between P_T_s and R_T_s included: HCC1: nonsilent mutations in *TP53* and *AKT2,* with activation of upstream gene signatures in *CTNB1, E2F1*and *TCF4* and suppression of *CDKN2A* upstream gene signature; HCC4: *CDH11* and *TGFBR3* stop codon mutations, DNA amplification in *POU5F1* and *UBD*, and DNA deletion in *GNAO1*with activation of *E2F1* and *TXB2* upstream gene signatures and suppression of upstream gene signatures *CDKN2A* and *RB1*; HCC5: DNA deletion in *CDH1* and a nonsilent mutation in *TP53* with activation of *E2F1* and *MYC* upstream gene signatures and suppression of upstream gene signatures *CDKN2A* and *TP53*; HCC11: activating mutation S45Y in *CTNNB1*, a stop codon mutation in *LRP1*, and a nonsilent mutation in the ANK2 domain of *CDKN2A*, with DNA amplification in *SOX9*. All four patients converge on shared inactivation of a key cell-cycle control tumor suppressor gene *CDKN2A* in P_T_s and R_T_s Using the same strategy to identify the most enriched pathways unique to R_T_s in all patients, cell cycle signaling was identified (Additional file 1: Table S7).

### HERC5 within chromosome 4q somatic CN deletion shows mRNA under-expression and predicts risk of survival and tumor recurrence in the primary tumors of HCC patients

Next we focused on the largest region of somatic CN deletion shared in both the P_T_s and R_T_s on chromosome 4q (~107 Mbp). Genes were evaluated within this region to identify a single molecular biomarker to predict HCC tumor recurrence and survival. WES and RNASeq from the four patients as well as RNASeq from P_T_s and P_NAT_s from a larger population of HCC Chinese patients who experienced tumor recurrence (n = 9 including the 4 described above) or did not (n = 12) were used to identified a predictor of tumor recurrence and survival. Using RNASeq data from the 21 P_T_s and P_NAT_s, DESeq-normalized log_2_ gene counts in the P_T_s were scaled by the matched P_NAT_s within each patient. Then these fold changes for each patient were used in a contrast between patients that experienced tumor recurrence (n = 9) and patients who did not (n = 12). A total of 273 genes were identified with |fold| >2 and p < 0.01 (Additional file 1: Table S9). To provide a large enough set of genes trending with under-expression in the recurrent patients, no multiple testing was implemented on these raw p-values. Further, the small sample sizes did not allow multiple testing corrections. Then genes within regions of DNA deletions in matched P_T_s and R_T_s in chromosome 4q (542 genes) and the 110 genes under-expressed (of the 273 genes both under- and over-expressed) in the P_T_s of HCC patients who experienced tumor recurrence (n = 9), relative to those who did not (n = 12; Additional file 1: Table S9) were intersected. From this analysis, the following genes were identified: *NAA11*, *HERC5*, *DDX60*, and *HERC6* which were evaluated individually for association with tumor recurrence using the 21 Chinese patient primary tumors.

**Fig. 3:**
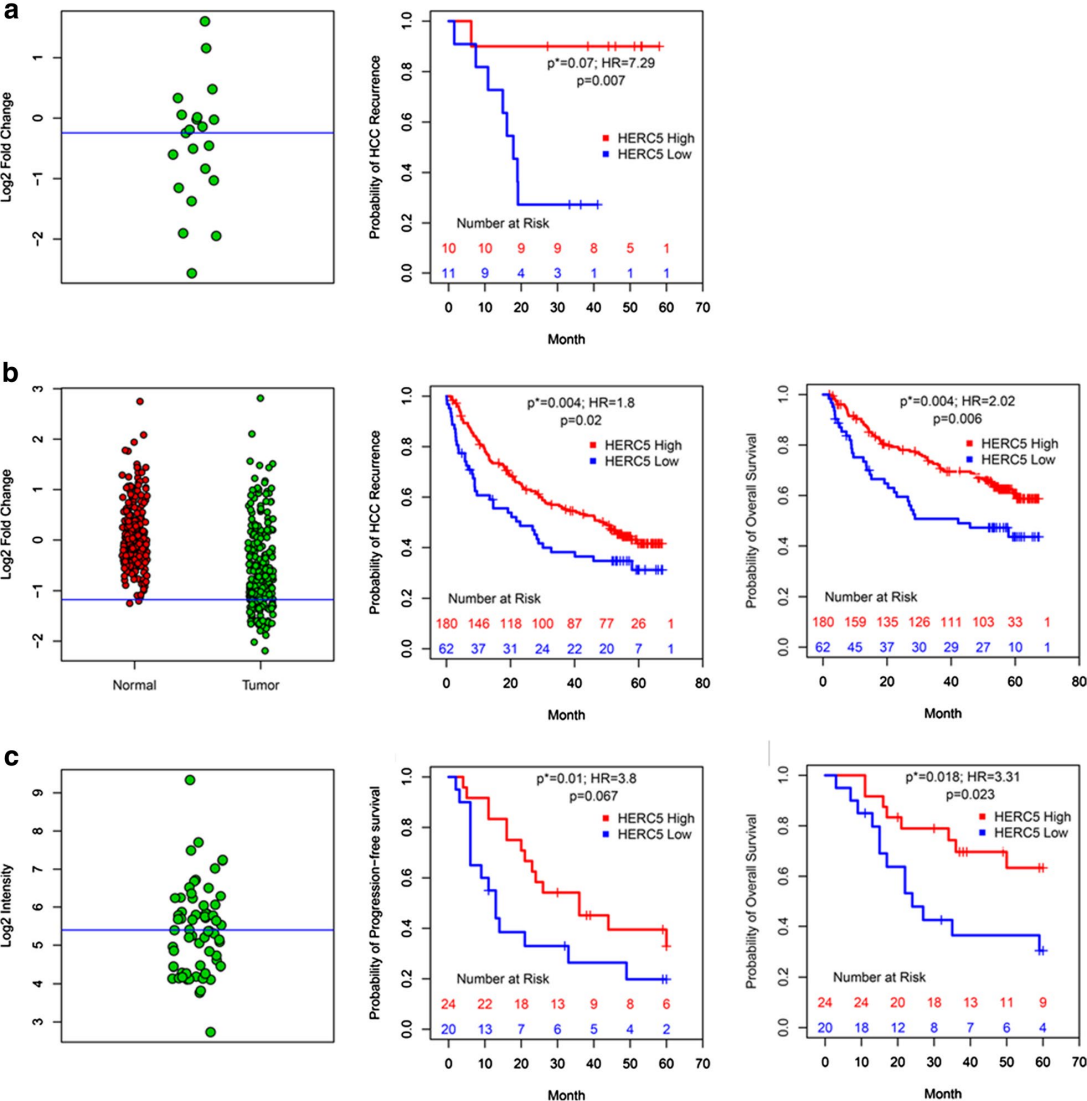
**a** *left HERC5* distribution of fold change values (log_2_ scale) for 21 Chinese patient primary tumors, *blue line* median of patients; *right* Kaplan–Meier (KM) curves comparing *HERC5* low (n = 11) to high expression (n = 10) predicting recurrence; **b** *left HERC5* distribution between normal liver (*red*; n = 239) and HCC tumors (*green*; n = 247; *19*), *blue line* mean(normals)-2SD; *middle* Kaplan–Meier (KM) curves comparing *HERC5* low (n = 62) to high expression (n = 180) predicting recurrence; *right* Same as *middle* predicting overall survival; **c** *left HERC5* distribution for HCC tumors, *blue line* median of 65 patients (*20*); *middle* KM curves comparing *HERC5* low (n = 20) to high expression (n = 24) predicting PFS; *right* Same as *middle* predicting overall survival. *p* log-rank test, p* Cox PH regression model, *HR* hazard ratio

For each of the four genes, the 21 Chinese patients were categorized into high/low gene groups using the median fold change expression (Fig. 3a). Kaplan–Meier log-rank tests indicated a significantly shorter time to recurrence in the gene *HERC5* low group (p = 0.007), while the other three genes did not significantly correlate with time to recurrence (p > 0.05), and after adjustment for HBV status pre-OLT, tumor grade, age, and gender, the trend remained for *HERC5* (p = 0.07; HR = 7.29 CI_95_ = [0.85, 62.62]; Fig. 3a; “Patients and methods”).

Two additional independent HCC datasets including primary tumors from patients who underwent resection were then evaluated [35, 36] where *HERC5* expression was categorized into high/low patient groups using healthy control liver tissue [36] or the median of the HCC patient distribution (no normal liver tissue was available in this study) [35]. After adjustment for cirrhosis status, gender, HBV/HCV status and TNM staging, *HERC5* was a significant predictor of overall survival (p = 0.004; HR = 2.02 CI_95_ = [1.26, 3.25]) and HCC recurrence (p = 0.004; HR = 1.80 CI_95_ = [1.20, 2.69]) in one dataset [36] with median time to recurrence in the *HERC5* low group = 23 months versus *HERC5* high group = 49 months (Fig. 3b; Table 2). Then in another independent HCC patient cohort [35], after adjustment for age, gender, and HBV status, *HERC5* under-expression was associated with shorter overall survival (p = 0.02; HR = 3.31 CI_95_ = [1.22, 8.96]) and PFS (p = 0.01; HR = 3.80 CI_95_ = [1.38, 10.43]) (Fig. 3c; Table 2). To verify that the lack of correlation between the other three candidate genes alternative to *HERC5* (i.e. *NAA11*, *DDX60*, and *HERC6*) and outcomes was not due to the small sample size used in the first patient dataset (n = 21), similar analyses were conducted on each gene independently using both larger HCC patient cohorts [35, 36] and no significant associations were observed for any of these three genes with any of the outcomes. An additional evaluation of specificity of the *HERC5* correlation with prognosis was conducted among all genes that were located within the deleted region of chromosome 4q using study [36] (Additional file 2). A total of 262 genes were both within the chromosome 4q region and present on the microarray from this study, of which 118 were under-expressed in the HCC tumor biopsies compared to tissue from the normal controls. A multivariate Cox PH model was calculated independently on each of the 118 genes, where each gene was cut into high or low groups based on the same criteria that was described previously (mean-2 SD of the normal liver distribution). *HAND2* had the most significant association with HCC recurrence, although it had a highly unbalanced distribution of high/low patients (3.6 % of patients in the low group). *HERC5* was the second most significant correlate with HCC recurrence (Additional file 3: Figure S8), demonstrating biological specificity of this gene independent of other genes with CN deletions in chromosome 4q.

In a separate study of HCV-positive cirrhosis patients, molecular subgroupings of patients were identified and shown to correlate with good versus poor prognosis [41]. *HERC5* was significantly over-expressed (p < 0.0001) in the good prognosis (n = 109) compared to poor prognosis group (n = 107) (Additional file 3: Figure S9).

### HERC5 loss induces CCL20 mRNA and protein and associates with FOXP3 positive Treg infiltration in HCC

**Fig. 4:**
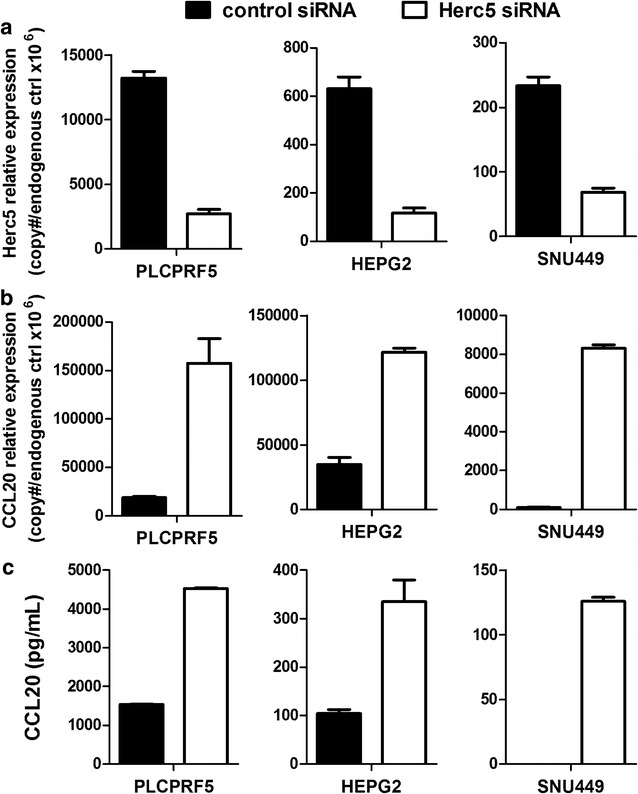
Up-regulated CCL20 expression after *HERC5* siRNA knockdown in HCC cell lines. **a** qRT-PCR analysis of *HERC5* mRNA expression in three HCC cell lines 96 h after transfection of negative control siRNA and *HERC5* siRNA. Shown is relative expression as copy numbers per 10^6^ endogenous control genes (average of expression levels of ACTB, GAPDH, and UBC); **b** qRT-PCR analysis of *CCL20* mRNA expression in three HCC cell lines 96 h after transfection of negative control siRNA and *HERC5* siRNA; **c** Secreted CCL20 in HCC cell culture medium was measured by ELISA 96 h after siRNA transfection. Standard deviations are represented for each *bar*

**Fig. 5:**
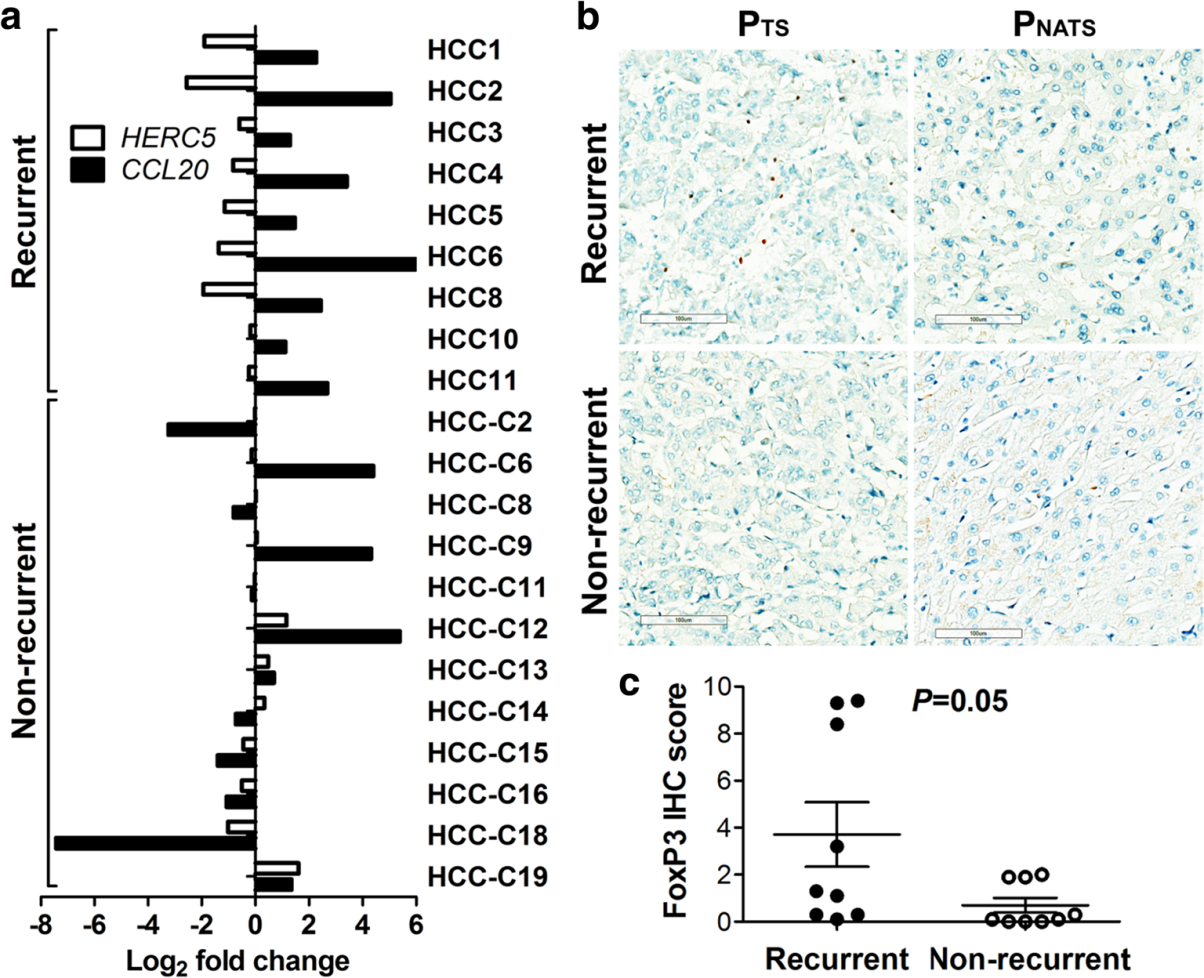
Negative correlation between *HERC5* and *CCL20* mRNA and the association of Tregs infiltration in P_T_s of recurrent patients. **a** Log_2_ fold changes *HERC5* and *CCL20* mRNA between and P_NAT_s in the patients who experienced tumor recurrence (n = 9) or the patients who did not (n = 12). A Student’s paired t-test between *HERC5* and *CCL20* log_2_ fold change values for the recurrent patients have p = 0.0003 and p = 0.49 for the non-recurrent patients. Negative correlation between *HERC5* and *CCL20* mRNA is present in the recurrent patients and not observed in the patients who did not recurrent. **b** Examples of FOXP3 IHC in P_T_s and P_NAT_s of a recurrent patient and a non-recurrent patient at ×20 magnification (530 × 460 µm in size per field). **c** FOXP3 IHC scores were calculated using the average numbers of FOXP3 positive stained lymphocytes in10 randomly selected fields at ×20 magnification (530 × 460 µm in size per field) in hepatocellular carcinoma for each sample. FOXP3 IHC score in P_T_s of recurrent patients (n = 9) were significantly higher than that in non-recurrent patients (n = 9) by Welch’s modified t-test (p = 0.05)

*HERC5* loss was next evaluated for biological significance in HCC. By studying the whole transcriptome expression profile of the HCC cell line PLCPRF5 with *HERC5* siRNA knockdown, *CCL20* was the most over-expressed transcript (fold = 5.8), and genes coding classic regulators of *CCL20* such as *TNF, NFKB1,* or *TRIM32* showed no change (Additional file 1: Table S10, Additional file 2). TaqMan qRT-PCR and ELISA assays confirmed the overexpression of CCL20 at the transcript and protein level with two additional HCC cell lines (SNU449 and HEPG2; Fig. 4). The difference of under-expression in *HERC5* in the recurrent patient P_T_s (mean = −0.3 fold) was significantly lower than that of P_T_s from patients who did not recur (mean = 1.1 fold; p = 0.001), confirming the results from the previous two array studies [35, 36] in this modest-sized study of Chinese patients. In addition, *HERC5* and *CCL20* mRNAs were significantly negatively correlated (p = 0.0003) in the P_T_s of HCC patients who experienced tumor recurrence and not in the P_T_s of HCC patients who did not experience tumor recurrence (p = 0.49; Fig. 5a). Immunostaining of FOXP3 in the primary tumors of the 21 HCC patients indicated significantly higher expression in the patients who experienced recurrence as compared to those who did not (Fig. 5c; p = 0.05).

## Discussion

Our results describe the genetic and genomic heterogeneity between patients with HCC and demonstrate clonal persistence in tumor recurrence post-OLT. A unique study design inclusive of P_T_s and R_T_s (plus normal adjacent tissue and recipient blood), with an integration of somatic SNVs, CNVs, and transcript profiling and well-defined phenotypic spectrum allowed us to elucidate key cancer driver genes and delineate those critical gatekeepers of cancer initiation and progression. This study is the first to show a clonal advantage in R_T_s post-OLT compared to P_T_s in HCC, where Wnt/β-catenin signaling activation and tumor suppressor gene *CDKN2A* inactivation occurs in both tumors.

DNA CN loss at chromosome 4q was identified as one of the only two hemizygous deleted regions shared between the primary and recurrent tumors in 3 of 4 Chinese HCC patients. This region accounts for ~107 Mbp in length and similar deletions have been associated with either poor prognosis or advanced disease stages in pancreatic, colorectal, non-small cell lung cancer (NSCLC), and HCC tumors [42–46]. Within NSCLC specifically, FISH assays identified the primary region of 4q21.2-22.1 to be associated with poor prognosis [44, 46, 47], and a recent study from this same group showed that hypermethylation of *HERC5* promoter (located at 4q22.1), and thus under-expression of the gene correlated with: positive disseminated tumor cells in the bone marrow, brain metastasis, and poor survival in both stage I adenocarcinoma and metastatic lung cancer patients [46]. Our results presented here in primary tumors of HCC patients are in agreement with these reports, underscoring the prognostic significance of *HERC5* under-expression, as we have demonstrated with microarray or RNASeq technologies in primary tumors of three independent cohorts of HCC patients.

Cancer cells can avoid an immune response by disabling components of the immune system—a process well known as immune evasion. These cells can paralyze activated immune effector cells such as infiltrating cytotoxic T lymphocytes (CTLs) and natural killer (NK) cells by secreting TGF-β or other immunosuppressive factors [48, 49] such as the cell-cycle control tumor suppressor gene *CDKN2A*. However, the genetic basis behind recruitment of inflammatory cells that are actively immunosuppressive, such as regulatory T cells (Tregs) and myeloid-derived suppressor cells (MDSCs) is largely unknown. CCL20 has been shown to be the only chemokine significantly up-regulated in HCC tissue [50], where it can selectively recruit Tregs to the tumor, contributing to an immunosuppressive tumor microenvironment and leading to poor prognosis in HCC [51, 52], Both the genetic basis and mechanism for the source of highly secreted CCL20 in HCC still remains to be elucidated. Our results suggest that loss of HERC5 is the genetic precursor for CCL20 over-expression and associates with increased Treg infiltration in HCC, one of the underlying mechanisms of immune evasion in HCC. Further, pathway analysis of the top induced genes (|fold| >2, 129 up-regulated/78 down-regulated) following knockdown of *HERC5* indicated activation of the IL17A pathway, as driven by over-expression of chemokine genes *CXCL13*, *CXCL15*, and *CXCL16*, with suppression of the Th2 cytokine IL-5, supported by the down-regulation of genes *TMF1*, *PDIA6*, *ELL2* and *APT1B1*. Elevated serum IL17 levels in primary HCC patients have been shown to correlate with risk of tumor recurrence following curative hepatectomy [53], while suppressed IL5 mRNA expression was correlated with poor survival in cervical cancer [54]. The modulation of such pathways when *HERC5* is silenced support an immunosuppressed environment for patients, thus influencing poor prognosis. HERC5 is an interferon-induced HECT-type E3 protein ligase that mediates type I interferon-induced ISGylation of protein targets; reduction in endogenous HERC5 blocks the IFN-induced ISG15 conjugation [55]. Thus host anti-viral responses are activated by the presence of HERC5 and reduced by its absence, suggesting an association with regulation of innate immune responses, a potential critical function leading to tumor recurrence in HCC. This hypothesis requires rigorous evaluation in future studies.

Unlike multiple transcriptomic studies that have developed gene signatures to predict tumor recurrence or survival in HCC [7–25], *HERC5* was not solely identified as a statistical correlate with outcome, rather, this gene is located within a large somatic copy number deletion on chromosome 4q in both P_T_s and R_T_s and was suppressed in HCC patients who experienced tumor recurrence, compared to those who did not. Unfortunately in this study, *HERC5* DNA loss was only evaluated in the 4 HCC patients with matched primary and recurrent tumor specimens, since DNA sequencing was not conducted on the remaining 17 HCC patients. Although, a recent study of 185 HCC patients showed that 28 % of patients’ primary tumors had a DNA deletion in *HERC5*, confirming a similar prevalence to patients with mRNA under-expression in this study (26 %; [56]; Additional file 3: Figure S9). Three independent studies with available comprehensive clinical and either RNASeq or microarray data were used to validate this clinical association totally 307 HCC patients. Taken together, these data confirm the robustness of our findings. *HERC5* is also not present in any gene signature among the 14 catalogued in the Liverome database [21].

Studies have shown varied levels of consistency for classifying patients with high risk for HCC recurrence using clinicopathologic variables such as tumor size, vascular invasion, tumor state, tumor grade, and alpha-fetoprotein levels and data presented here suggest a single transcript as an important variable to couple to these relevant clinical factors for identifying HCC candidates for OLT. A single transcript also provides a pragmatic diagnostic assay for clinical use.

In a disease with no established adjuvant treatments, substantial shortage of donor organs, and high financial burden, identifying eligible patients for OLT with low recurrence risk at the molecular level accompanied by current clinical criteria, has potential to significantly improve patient clinical outcomes. This study is a first in HCC using comprehensive genetics and genomics patient profiling supported by large independent patient cohorts to provide evidence for such a biomarker and characterize the shared genetic drivers between primary and recurrent HCC. We believe that the translational study design and analytical strategy presented in this work will inspire other genetics studies beyond HCC recurrence and into other primary malignancies where metastases to other organs systems are observed.

## Conclusion

This study provides evidence for a clonal selection advantage in the recurrent tumor, as compared to primary tumor in HCC patients and Wnt/β-catenin signaling was identified as the most regulated oncodriver pathway in matched primary and recurrent tumors. *HERC5* was also identified as a prognostic biomarker for both survival and tumor recurrence in HCC patients in three independent HCC patient cohorts. Genetic- and genomic-driven under-expression of this gene is associated with *CCL20* induction, suggested to increase Treg infiltration and ultimately poor prognosis in HCC patient post-OLT.

## Additional files

10.1186/s12967-015-0743-2 Supplementary Methods.
10.1186/s12967-015-0743-2 Supplementary Figures.
10.1186/s12967-015-0743-2 Supplementary Tables.

## Abbreviations

HCC: hepatocellular carcinoma
P_T_: primary tumor
P_NAT_: primary normal adjacent tissue
P_B_: recipient blood
R_T_: recurrent tumor
R_NAT_: recurrent normal adjacent tissue
HBV: hepatitis B virus
HCV: hepatitis C virus
OLT: orthotopic liver transplantation
WES: whole exome sequencing
RNASeq: RNA sequencing
Vf: variant frequency
SNV: single nucleotide variant
indel: insertion or deletion
CNV: copy number variation
HR: hazard ratio
CI: confidence interval
UCSF: University of California San Francisco
CR: clonal relationship value
WGS: whole genome sequencing
Qphred: Phred sequence value score
VCF: variant call format
LRR: Log_2_ R ratios
BAF: B-allele frequency
CTLs: cytotoxic T lymphocytes
NK: natural killer
Tregs: regulatory T cells
MDSCs: myeloid-derived suppressor cells
HERC5: HECT And RLD Domain Containing E3 Ubiquitin Protein Ligase 5
CCL20: chemokine ligand 20
