# Supplementary material for: HERC5 is a prognostic biomarker for post-liver transplant recurrent human hepatocellular carcinoma
Source: J Transl Med. 2015 Dec 11;13:379. doi: 10.1186/s12967-015-0743-2 (PMC4676172; doi:10.1186/s12967-015-0743-2)
Supplement: Supplementary file 2 — 10.1186/s12967-015-0743-2 Supplementary Figures. [file 12967_2015_743_MOESM2_ESM.docx]

**Supplementary Methods**

***DNA sequence read mapping and variant calling (continued)***

All sequence data was QC’d for read counts, quality values, kmer usage, GC-content, and all other relevant parameters within FastQC (v0.10.1). The DNA sequence was aligned to the human genome (UCSC hg19; Feb 2009 release; Genome Reference Consortium GRCh37) using Bowtie2 (v2.0.0-beta7; *1*) and both indel realignment and PCR duplicate removal was conducted using GATK (v2.3.4; *2*) and Picard (v1.85), respectively. All mapping summaries for each specimen from WES are provided in **Supplementary Table 2**. For RNASeq and WES data, SAMtools (v0.1.18; *3*) mpileup was used (Qphred>30 and mapping quality>30) with VarScan2 (v2.3.2; *4*) to call both single nucleotide variants (SNVs) and insertion/deletions (indels) relative to the human reference with parameters: variant minimum frequency>5%, VarScan2 p-value for variant calls (based on Fisher’s exact test) <0.01, minimum coverage=20, minimum reads=3, minimum average quality=30, minimum frequency for homozygote=0.8. For WGS data, where depth was much lower than WES data, the following arguments were used: minimum coverage=15, minimum reads=2, minimum average quality=13, Qphred>13 and mapping quality>1.

Variant calls were made for all samples in a batch call and exported as a Variant Call Format (VCF) (v4.1) file. Then variant call refinement was conducted according to the following rules for somatic SNV and indel calls between pairs of P_T_ vs. P_B_, P_NAT_ vs. P_B_, R_T_ vs. P_B_, and R_NAT_ vs. P_B_, where P=primary, R=recurrent, T=tumor, NAT=normal adjacent tissue, and B=blood as follows:

1. Average Qphred of reference or variant (or both) >30 for WES (13 for WGS) in normal and tumor,
2. P-value from Fisher’s exact test of read counts between normal/tumor and reference/variant<0.01,
3. Variant frequency (Vf) in normal<3%, and
4. Tumor Vf at least 5% greater than Vf of normal.

To identify somatic SNVs specific to both the P_T_ and R_T_ samples for each patient, those SNVs identified between P_NAT_ vs. P_B_ that were shared with the list of P_T_ vs. P_B_ SNVs were removed, as were those SNVs identified between R_NAT_ vs. P_B_ that were shared with the list of R_T_ vs. P_B_ SNVs. Annotation and variant effects on genes was then conducted using ANNOVAR (v2013-07-28; *5*) and all nonsilent (i.e. nonsynonymous, stopgain, or stoploss) SNVs or nonsilent indels (i.e. frameshift substitution) were selected based on:

1. Either an unknown or minor allele frequency (MAF)<0.05 in the 1000 genomes with all races, and
2. Not located within either tandem repeat or disruptive repeat regions from the UCSC Genome database (http://genome.ucsc.edu).

All silent SNVs and indels were selected based on predicted exon function (synonymous) and criterion #2 above. Somatic SNV and indel counts for each patient are provided in **Supplementary Table 3**. Any SNVs identified in either the WGS or RNA sequencing, using this similar stringent selection criteria are provided in **Supplementary Table 4** with the associated Vf values in both tumor and either blood or normal adjacent tissue. Somatic indels are provided in **Supplementary Table 5.**

Germline SNV calls were also refined for the pairs described above according to the following rules:

1. Average Qphred of reference or variant (or both) >30 for WES (13 for WGS) in normal and tumor,
2. P-value from VarScan2 Fisher exact test (relative to reference genome) <0.01 in both normal and tumor, and
3. Minimum Vf in both normal and tumor > 5%.

All nonsilent (i.e. nonsynonymous, stopgain, or stoploss) SNVs or nonsilent indels (i.e. frameshift substitution) were selected based on:

1. Either an unknown or minor allele frequency (MAF)<0.05 in the 1000 genomes with all races, and
2. Not located within either tandem repeat or disruptive repeat regions from the UCSC Genome database (http://genome.ucsc.edu).

This provided the germline SNV list.

***Specimen sequencing summary***

To characterize the genetic differences between the primary and recurrent tumors in HCC, WES was conducted on the P_T_, R_T_, P_NAT_, R_NAT_, and P_B_ from four recurrent patients (20 total specimens), RNASeq on the P_T_, R_T_, P_NAT_, R_NAT_, and WGS on these P_T_s to validate certain single nucleotide variants (SNVs; **Table 1 and Supplementary Table 1**). The mean WES depth for the 20 specimens ranged from 66-109X (mean=92.1X) and an average of 73.4% coverage (Qphred>30) was observed at a minimum of 10X depth (**Supplementary Table 2**). RNASeq was also conducted only on P_T_s and P_NAT_s in the remaining 17 patients. WES and RNASeq data have been deposited into dbGaP (phs000782.v1.p1) and GEO (GSE56545).

***Patient identity QC***

To verify the identity of the P_T_. P_B_, and P_NAT_, as well as the R_T_ and R_NAT_, a selection of 300 heterozygous single nucleotide polymorphisms (SNPs) with MAFs>0.3 and <0.7 were selected from the 1000 genomes database. Then all five DNA samples (four samples for RNA) per patient were clustered to observe any major discrepancies in subject or specimen labeling (**Supplementary Figures 2A-B**). Additionally, the germline variants called for both the primary and recurrent tumor samples were clustered in heatmap to verify the appropriate blocking of variant calls within a quintet of DNA specimens for each of the 4 patients (**Supplementary Figure 2**).

***Clonal relationship (CR) value***

The clonal relationship of each primary and recurrent tumor pair was assessed by enumerating all nonsilent SNVs with a PolyPhen designation of medium or high for each tumor independently.  The number of matched locus variants that were in-common between the two matched subject tumors was determined and the ratio of shared variants relative to the total number of variants identified for each tumor type was calculated. Subjects with CR values greater than 0.5 were considered to be of recipient origin (**Supplementary Figure 5**).

***Donor presence within the recurrent tumor***

Under the assumption that the incidence of donor-transmitted malignancy is extremely low (<0.05%), the cancer clonal composition of the donor tumor biopsy should consist largely of the primary tumor (*6*). However, it is difficult to obtain pure recipient tumoral biopsy from the donor tissue using either radiofrequency ablation (HCC1, HCC4, and HCC5) or video-assisted thorascopic surgery (HCC11) techniques, as was conducted in these patients. To identify the proportion of primary tumor (recipient-based) within the donor liver biopsy, two independent calculations were conducted. The first calculation involved regressing the Vfs of all somatic (nonsilent and silent) variants called in the recurrent tumor versus those in the primary tumor. The somatic variants unique to either the primary tumor or recurrent tumor were removed to not bias the best fit line. The slope of this curve was then calculated with least squares and subtracted from 1 to indicate a ‘donor tissue contamination’ proportion in the primary tumor (**Supplementary Figure 6A**).

The second approach utilized a set of recipient-unique homozygous SNPs and the contamination of donor cells was calculated as the average(1- Vfs of the recipient-unique homozygous alleles in the recurrent tumor), where the alleles affected by CNVs or located in chr X/Y were excluded.  The recipient-unique homozygous alleles are those alleles with >90% Vf in the recipient primary blood and <10% Vf in the donor, specific for each patient (**Supplementary Figure 6B**).

***Somatic copy number variation (CNV) analysis***

R packages DNAcopy and ExomeCNV were used to identify CNVs using the read depth derived from WES alignments. A 1Mb window size was selected to identify CN segments for three reasons: 1) to identify large-scale segments rather than regions of focal variation, 2) coding region gene structure can be hundreds of kilobases long in exome capture, requiring a large window to encompass entire genes, rather than parts of a gene, and 3) a summary across all patients showed that <3% of segments were excluded at a 1Mb threshold. Log_2_ R ratios (LRR) of CNVs were calculated across the genome as the difference between all tumor and normal comparisons and heterozygous or homozygous assessments were determined for each DNA segment. CNV results are provided in **Figure 1.**

***Integrated pathway analysis***

Pathway analyses were conducted using Ingenuity Pathway Analysis (IPA; Ingenuity, Redwood City, CA); plots and additional analyses were conducted in R (v3.0.1; R: A Language and Environment for Statistical Computing, R Development Core Team, R Foundation for Statistical Computing, Vienna, Austria, 2013). Two types of pathway analyses outputs were conducted. The first associated the upstream genetic effects on pathway regulation using all genes harboring nonsilent somatic SNVs and indels shared between the P_T_s and R_T_s for a patient with medium-to-high PolyPhen functional effects, combined with genes within CNV segments of significant amplification or deletion AND gene expression support |fold|>2 (P_T_/P_NAT_ and R_T_/R_NAT_); p-values from DESeq <0.05. No multiple testing adjustment was applied for reasons consistent with the explanation provided in the ***RNASeq*** methods section. These genes were then used in canonical pathway enrichment analysis using IPA. The comparison tool in IPA was used to combine all pathway information from each of the four patients and a composite score was computed per pathway as the summation of the –log_10_(pvs) across the patients for a pathway (**Supplementary Table 6; Supplementary Figure 7**). This same pathway enrichment analysis was also conducted for those genes using the same criteria for just those events unique to the R_T_s across all four patients (**Supplementary Table 7**). The second pathway analysis output included the upstream analysis output from IPA with input of differentially expressed genes (shared between P_T_ vs. P_NAT_ and R_T_ vs. R_NAT_; |fold|>2 and p<0.01) to identify transcription factors (TFs) most enriched by genes regulated by the TF. This is referred to as an upstream gene signature (**Supplementary Table 8**).

***Specificity of HERC5 prognostic correlation among genes in chr4q***

To demonstrate prognostic specificity of *HERC5* compared to other genes within the same DNA deletion region in chromosome 4q, a similar time-to-event analysis was conducted on each of the genes found within this deleted region from one of the independent studies (*14*). Of the 262 genes identified on 4q and represented on the array, 118 were under-expressed in the tumor biopsies compared to tissue from the normal controls. Using these 118 genes in independent time-to-event models, *HAND2* had the most significant association with HCC recurrence, although it had a highly unbalanced distribution of high/low patients (3.6% of patients in the low group). *HERC5* was the second most significant correlate with HCC recurrence (**Supplementary Figure 8**), demonstrating biological specificity of this gene independent of other genes with CN deletions in chromosome 4q.

***HERC5 siRNA transfection experiments***

SiRNA transfections were carried out using Lipofectamine RNAiMAX (Life Technologies). siRNA targeting Herc5 was supplied by Dharmacon Inc, and the sequence is GGAAGUAGCAUAACUGUCAUU. AllStars Neg. Control siRNA (Qiagen) was used as negative control for transfection. Both control siRNA and Herc5 siRNA were transfected at a final concentration of 50nM. Culture medium was replaced with fresh medium at 48 hour after transfection. Cell culture supernatant and total RNA were prepared at 96 hour for Microarray study, qRT-PCR and ELISA.

***Microarray study of HERC5 siRNA knockdown in HCC cell lines***

Total RNA was extracted from HCC cell lines transfected with *HERC5* siRNA or negative control siRNA using the ZR RNA MicroPrep kit (Zymo Research, Orange, CA). RNA purity and concentration were determined spectrophotometrically (260/280>1.9). RNA quality was assessed on an Agilent 2100 Bioanalyzer using the RNA 6000 Nano LabChip®

Generation of biotin-labeled amplified cRNA from 75 ng of total RNA was accomplished with the MessageAmpTM Premier RNA Amplification Kit (Ambion, Austin, TX). The concentration and purity of the cRNA product were determined spectrophotometrically. Fifteen micrograms of each biotin-labeled cRNA was fragmented for hybridization on Affymetrix Human Genome U133 Plus 2.0 GeneChip® arrays. All GeneChip® washing, staining, and scanning procedures were performed with Affymetrix standard equipment. Data capture and initial array quality assessments were performed with the GeneChip Operating Software (GCOS) tool. Stratagene's (La Jolla, CA) ArrayAssist® Lite software was used to calculate probe-level summaries (GC-RMA and MAS5) from the array CEL files.

***qRT-PCR (TaqMan) Validation***

The primers and probes of TaqMan gene expression assays CCL20 (Hs01011368_m1), *HERC5* (Hs00180943_m1), *ACTB* (Hs99999903_m1), *GAPDH* (Hs99999905_m1) and *UBC* (Hs00824723_m1) were purchased from Life Technologies. cDNA samples in panel 1 were preamplified using TaqMan Pre-Amp Master Mix (Life Technologies, CA), according to the manufacturer’s instructions. Reactions contained 5 μL of cDNA, 10 μL of Pre-Amp Master Mix, and 5 μL of 0.2× gene expression assay mix (comprised of all primer/probes to be assayed) at a final reaction volume of 20 μL. Reactions were cycled with the recommended 14-cycle program and then diluted 1:5 with TE buffer. Preamplified cDNA was used immediately or stored at 20°C until processed. For PCR, the BioMark RT-PCR System (Fluidigm, CA) was utilized as previously described (*9*). The experiments were run in triplicate. Average Cycle Threshold (Ct) values were used to determine sensitivity and specificity of the designed probes. Ct values were extracted from each assay with the SDS v2.0 software tool (Applied Biosystems, CA). The average Ct values of all available reference gene assays within a sample were utilized **(Figure 4).**

***Cell culture and ELISA assay***

HCC cell lines were grown in RPMI1640 medium supplemented with 10% fetal bovine serum. Human CCL20/MIP-3 alpha Quantikine ELISA Kit (R&D Syatems, DM3A00) was used to quantify secreted CCL20 in cell culture medium. Assays were carried out following product manual and using 100μL supernatant from 5x10^3^ cells cultured in 96-well plate after 96h siRNA transfection **(Figure 4).**

***FoxP3 Immunochemistry assays***

The FoxP3 IHC assay was performed on Ventana Discovery Ultra (Ventana Medical Systems, AZ, USA). Samples were firstly deparaffinized under 69°C for 16 minutes, and then pre-treated with Cell Conditioning 1 for 36 minutes at 95°C. Sections were then incubated with 1:25 diluted FoxP3 rabbit monoclonal antibody (M3974, Spring Bioscience, CA, USA) for 32 minutes at 37°C. Secondary antibody (OmniMap anti-rabbit HRP, Ventana Medical Systems, AZ, USA) was then added and sections were incubated for 8 minutes. IHC signals were then visualized by incubation with ChromoMap DAB kit (Ventana Medical Systems, AZ, USA) for 4 minutes. Sections were then counterstained with hematoxylin, dehydrated in grades of ethanol and xylene, and mounted with coverslips.FoxP3 positive stained lymphocytes were counted by a pathologist in hepatocellular carcinoma and adjacent no tumor areas, respectively for comparison average number of positive lymphocytes were counted in randomlyselected 10 fields at 20 X magnification (530X460 µm in size per field).

**References**

1. Langmead B, Salzberg SL. Fast gapped-read alignment with bowtie 2. Nat Methods 2012; **9**(4): 357-359.

2. McKenna A, Hanna M, Banks E, Sivachenko A, Cibulskis K, Kernytsky A, Garimella K, et al. The genome analysis toolkit: A MapReduce framework for analyzing next-generation DNA sequencing data. Genome Res 2010; **20**(9): 1297-1303.

3. Li H, Handsaker B, Wysoker A, Fennell T, Ruan J, Homer N, Marth G, et al. The sequence Alignment/Map format and SAMtools. Bioinformatics 2009; **25**(16): 2078-2079.

4. Koboldt DC, Zhang Q, Larson DE, Shen D, McLellan MD, Lin L, Miller CA, et al. VarScan 2: Somatic mutation and copy number alteration discovery in cancer by exome sequencing. Genome Res 2012; **22**(3): 568-576.

5. Wang K, Li M, Hakonarson H. ANNOVAR: Functional annotation of genetic variants from high-throughput sequencing data. Nucleic Acids Res 2010; **38**(16): e164.

6. Kim B, Woreta T, Chen PH, Limketkai B, Singer A, Dagher N, Cameron A, et al. Donor-transmitted malignancy in a liver transplant recipient: A case report and review of literature. Dig Dis Sci 2013; **58**(5): 1185-1190.

7. Sung WK, Zheng H, Li S, Chen R, Liu X, Li Y, Lee NP, et al. Genome-wide survey of recurrent HBV integration in hepatocellular carcinoma. Nat Genet 2012; **44**(7): 765-769.

8. Jiang Z, Jhunjhunwala S, Liu J, Haverty PM, Kennemer MI, Guan Y, Lee W, et al. The effects of hepatitis B virus integration into the genomes of hepatocellular carcinoma patients. Genome Res 2012; **22**(4): 593-601.

9. Huang J, Morehouse C, Streicher K, Higgs BW, Gao J, Czapiga M, Boutrin A, et al. Altered expression of insulin receptor isoforms in breast cancer. PLoS One 2011; **6**(10): e26177.
