# Supplementary material for: HERC5 is a prognostic biomarker for post-liver transplant recurrent human hepatocellular carcinoma
Source: J Transl Med. 2015 Dec 11;13:379. doi: 10.1186/s12967-015-0743-2 (PMC4676172; doi:10.1186/s12967-015-0743-2)
Supplement: Supplementary file 3 — 10.1186/s12967-015-0743-2 Supplementary Tables. [file 12967_2015_743_MOESM3_ESM.docx]

**Supplementary Figures**


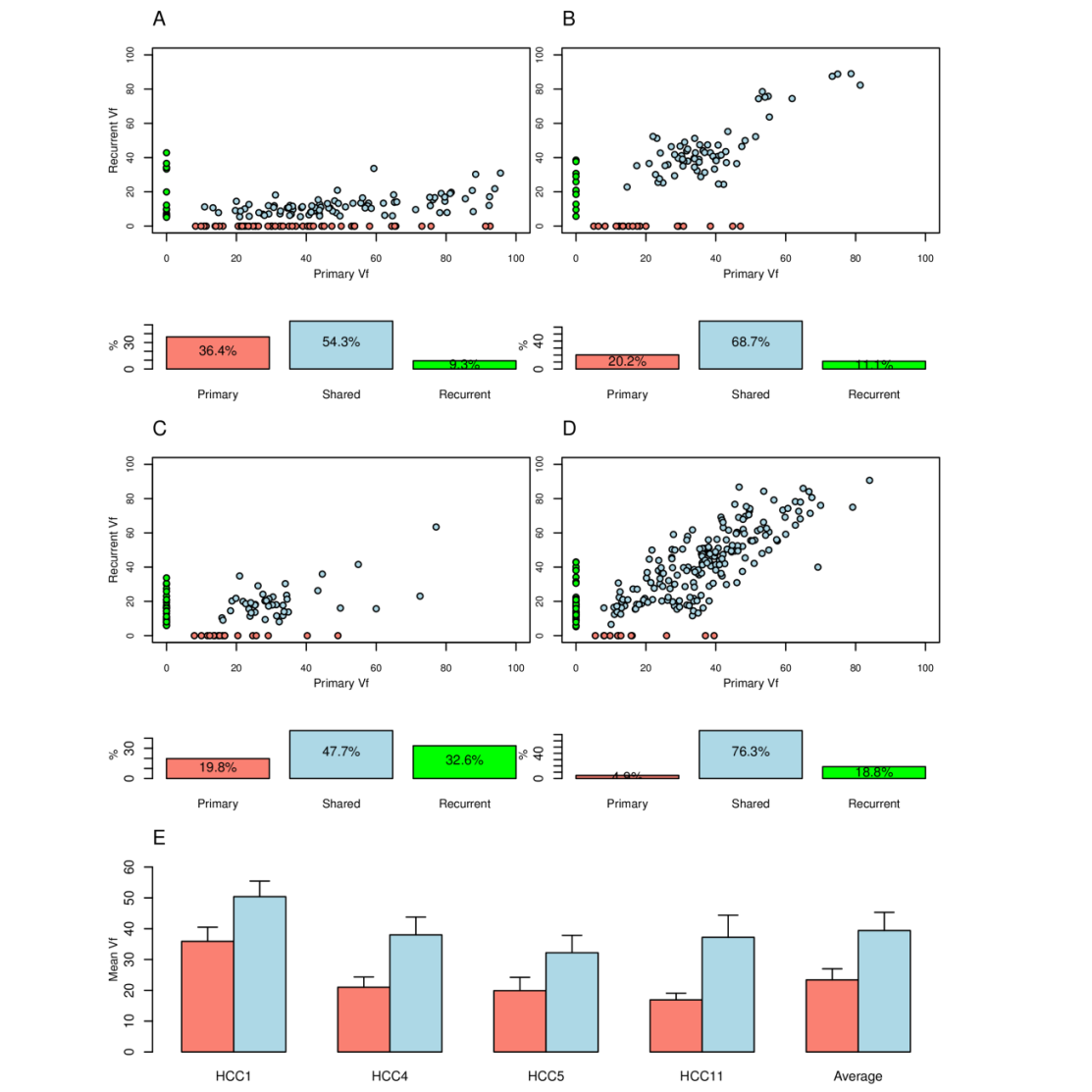


**Supplementary Figure 1:** Clonal advantage in recurrent tumors. **A-D**) Non-silent somatic SNV variant frequencies (Vfs) between the recurrent tumor (y-axis) and primary tumor (x-axis) (top scatter plots) and bottom barplots showing the proportion of counts unique to the primary tumor (salmon), shared between the primary and recurrent tumors (light blue), or unique to the recurrent tumor (green) for: **A**=HCC1, **B**=HCC4, **C**=HCC5, and **D**=HCC11. **E**) Non-silent somatic SNVs shared between primary and recurrent tumors (blue bars) show clonal advantage compared to those unique to primary tumors (salmon bars) in the 4 HCC patients and the average across all four patients. Error bars represent 95% confidence intervals. Vf=variant allele frequency; primary=primary tumor; shared=common to both primary and recurrent tumor; recurrent=recurrent tumor.


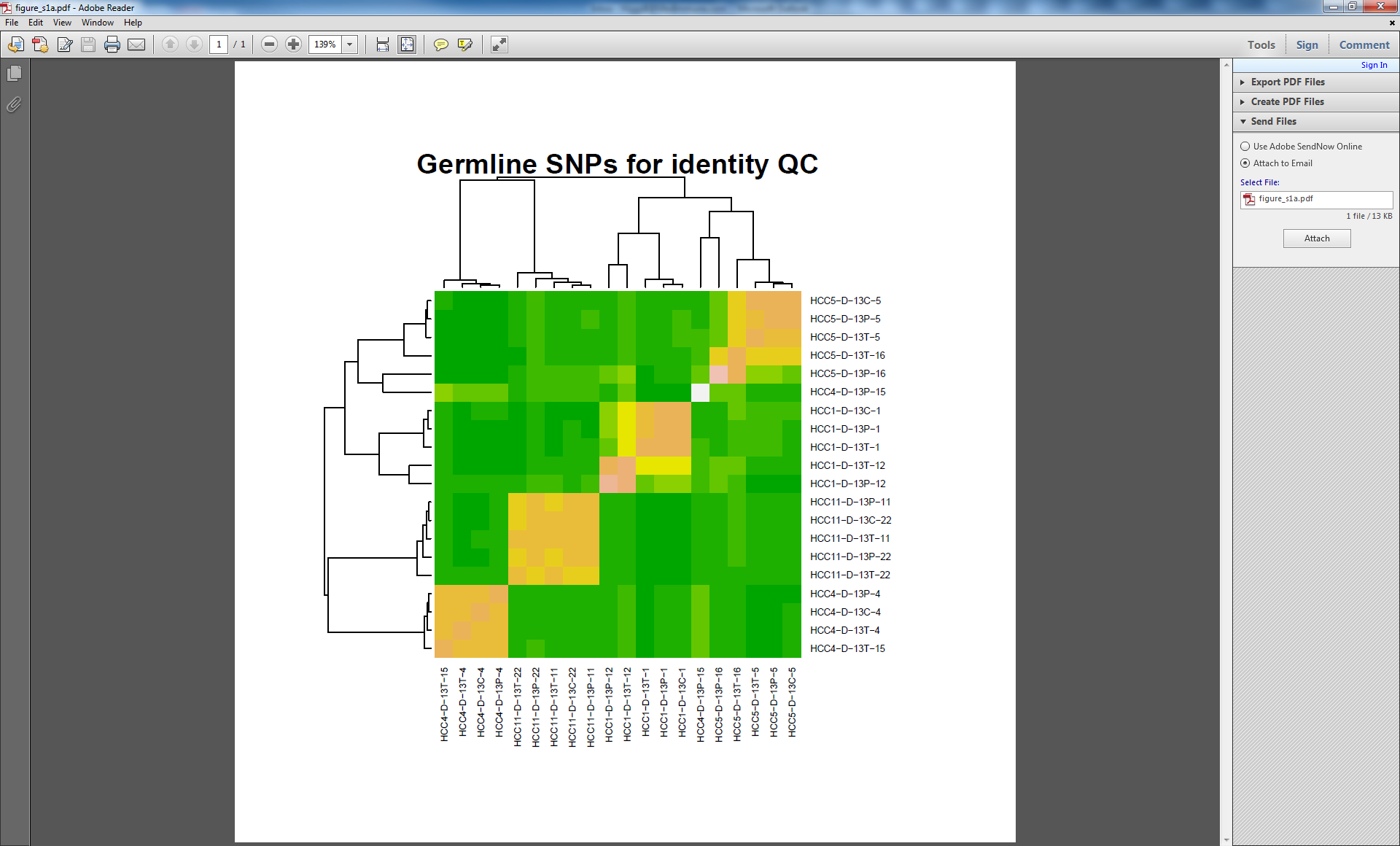

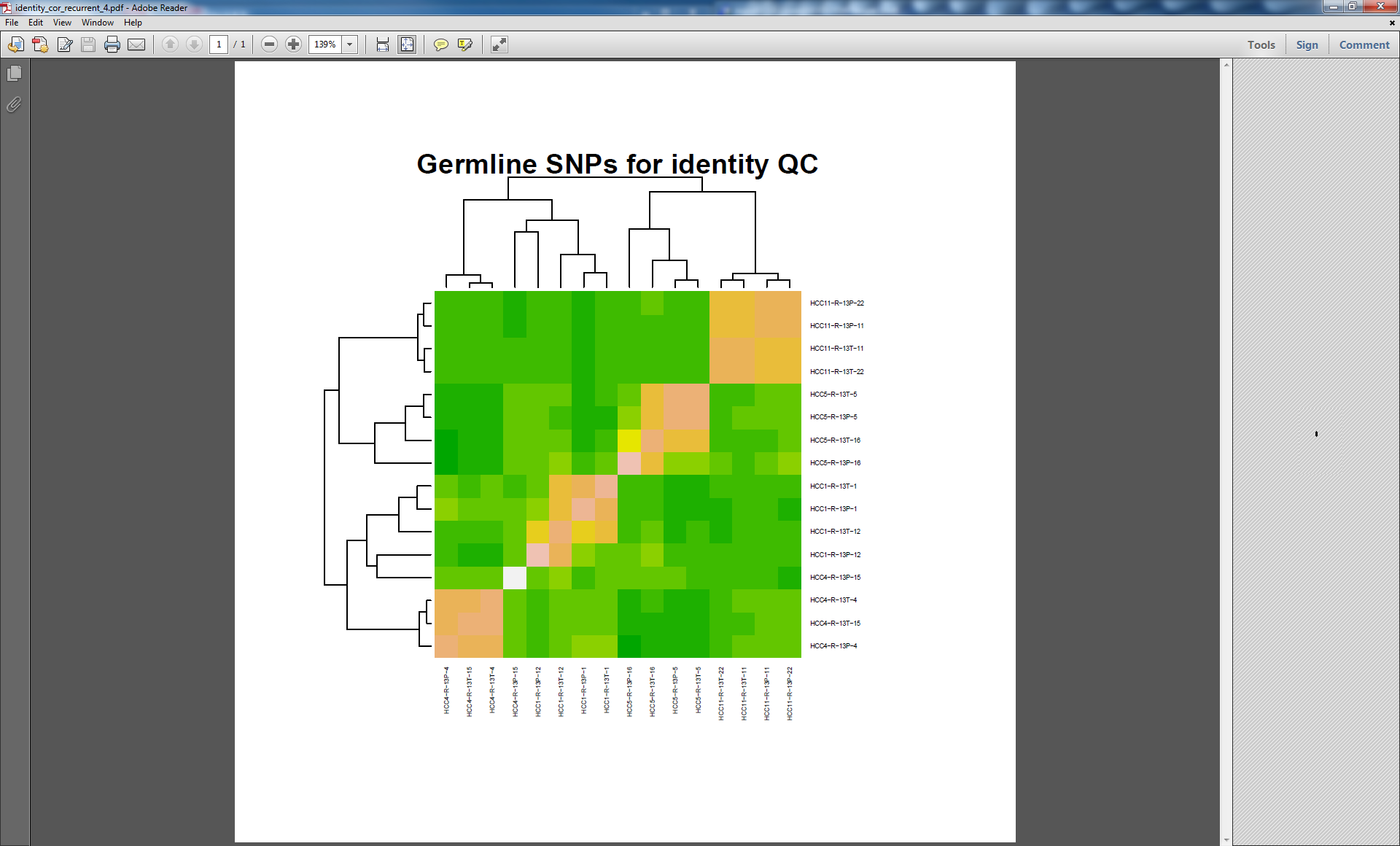


**A**

**B**

**Supplementary Figure 2:** **A)** WES DNA germline SNP correlation heatmap of 5 specimens (primary tumor, recipient normal adjacent tissue, recurrent tumor, donor normal adjacent tissue, and recipient blood) per patient using 300 heterozygous SNPs identified from the 1000 genomes project database; and **B)** RNA correlation heatmap for the same patients, without the recipient blood specimens. Brown/yellow/white blocks and cluster dendrograms on each axis indicate similarity in groupings of each specimen quintet for DNA and quartet for RNA.


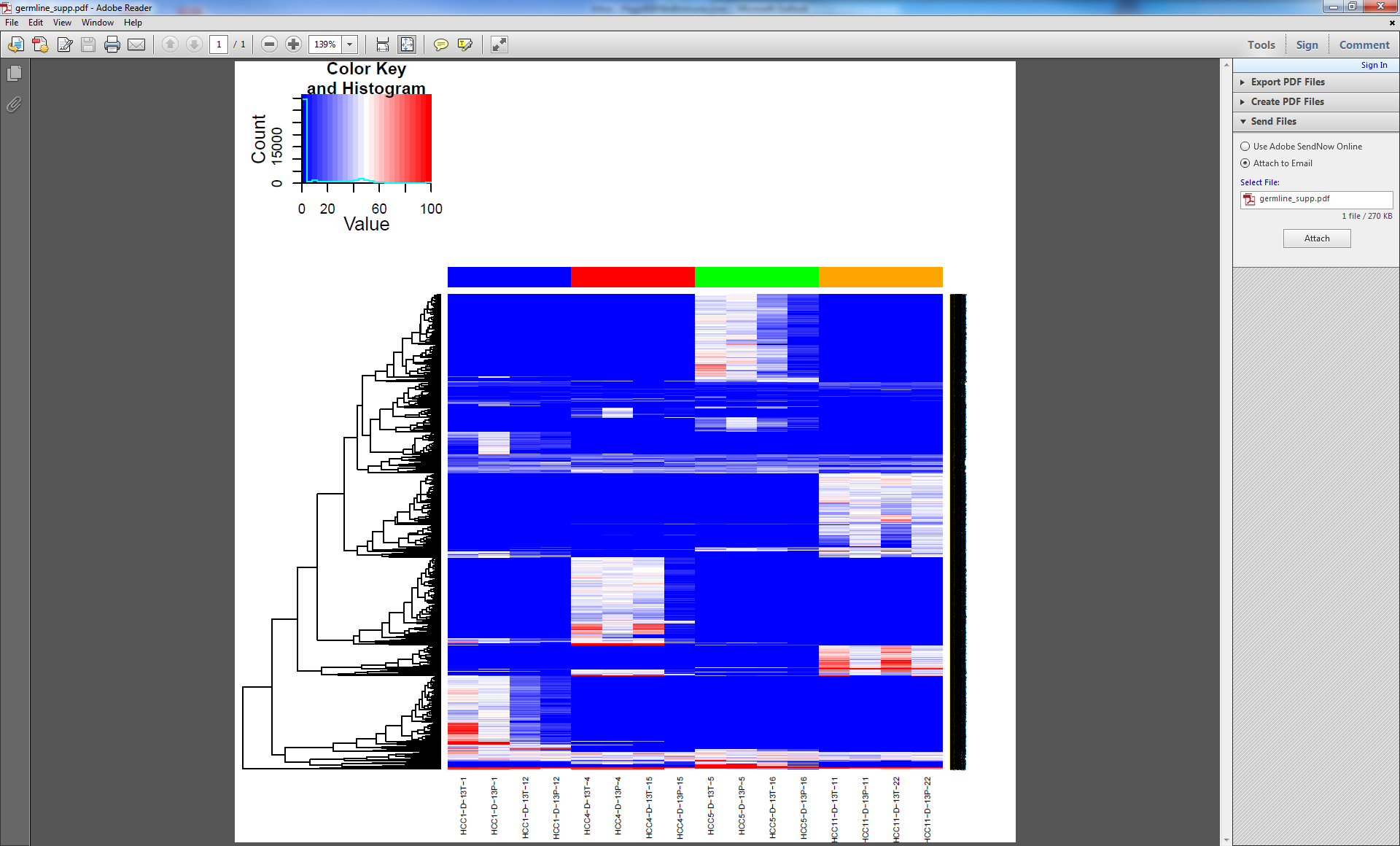


**Supplementary Figure 3:** WES DNA germline SNVs heatmap of 5 specimens (primary tumor, recipient normal adjacent tissue, recurrent tumor, donor normal adjacent tissue, and recipient blood) per patient.

**
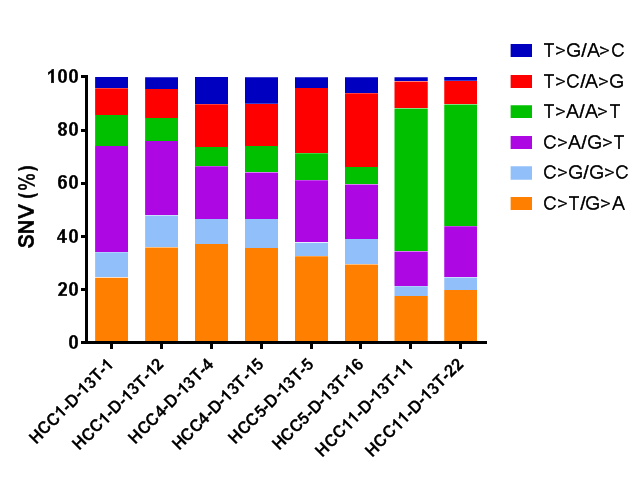
**

**Supplementary Figure 4:** WES somatic SNV transition/transversion patterns in the primary and recurrent tumors of four HCC patients.

**Supplementary Figure 5:** Determination of tumor origin using a derivation of the clonal relationship value calculation and all non-silent SNVs with medium-to-high polyphen predictions.

**A**


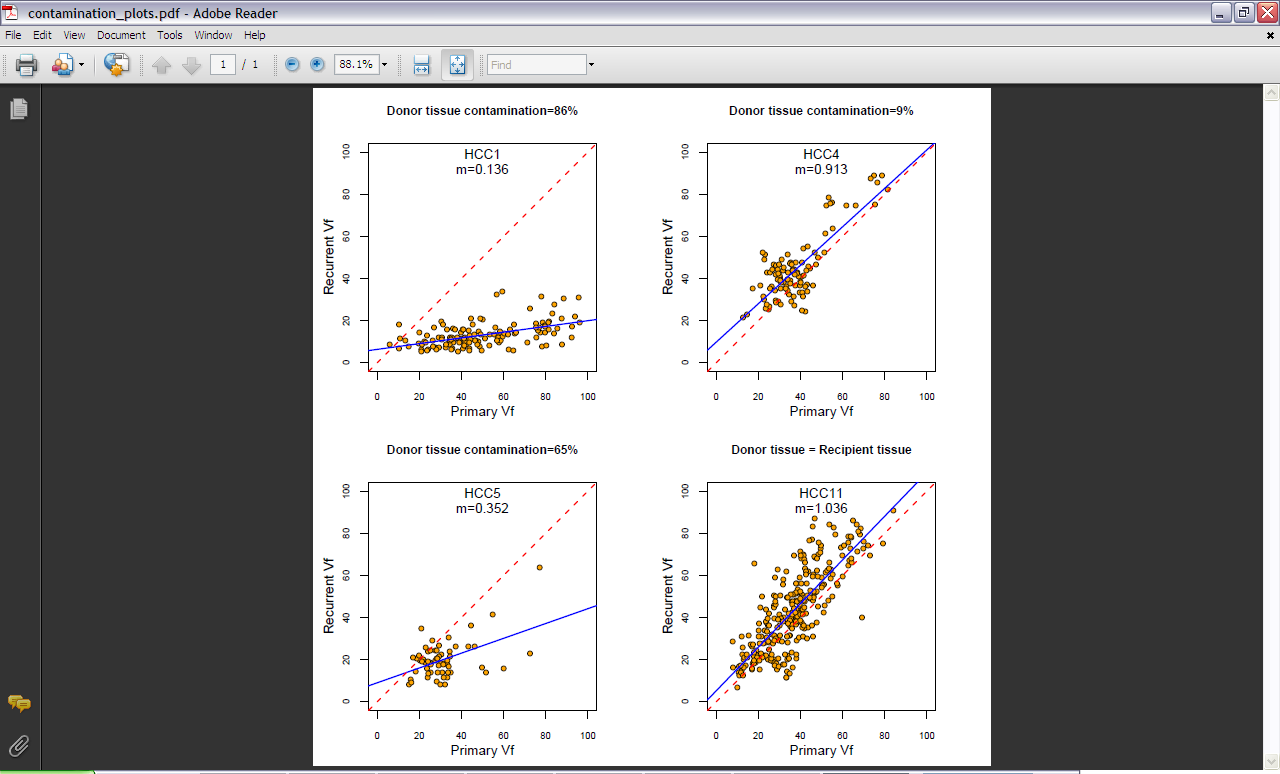


**B**


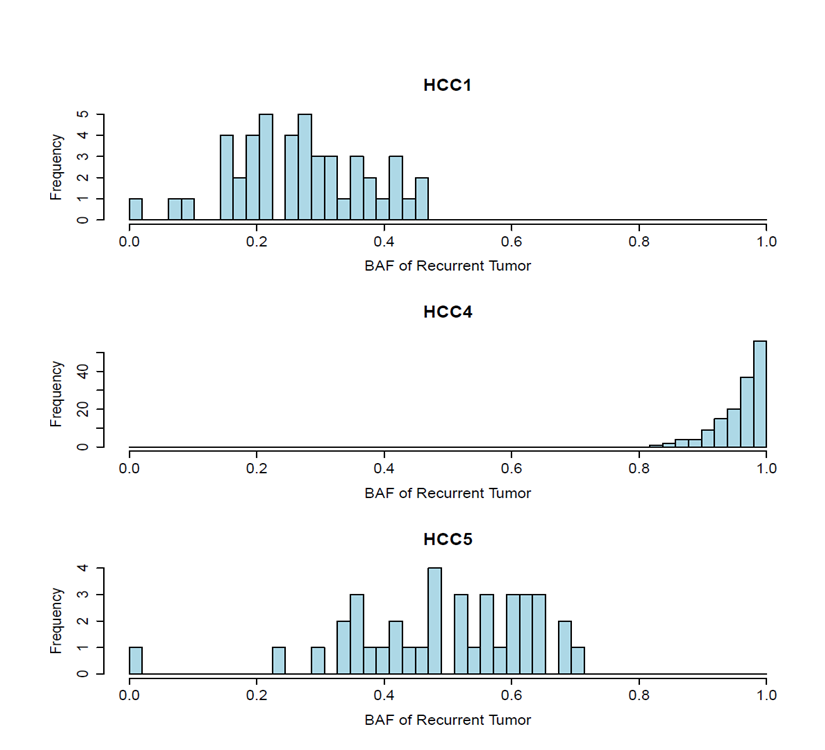


**Supplementary Figure 6:** Donor contamination calculations using **A**) the slope (m) of all somatic SNV Vfs between the recurrent tumor (y-axis) and primary tumor (x-axis) for HCC1, HCC4, HCC5, and HCC11. All somatic variants unique to either primary tumor or recurrent tumor have been removed from the plots, as have any Vf<5% in either the primary or recurrent tumor samples. Red dashed line indicates x=y and solid blue line indicates the least squares fit. **B**) B-allele frequency (BAF) histograms using recipient-unique homozygous single nucleotide polymorphisms (SNPs) for HCC1, HCC4, and HCC5. After removal of all alleles affected by CNVs, the contamination of donor cells was calculated as the median(1- Vfs of the recipient-unique homozygous alleles in the recurrent tumor). HCC11 primary and recurrent tumors are of recipient-origin, so that sample is not included here. See **Supplementary Methods** for more details on calculation.


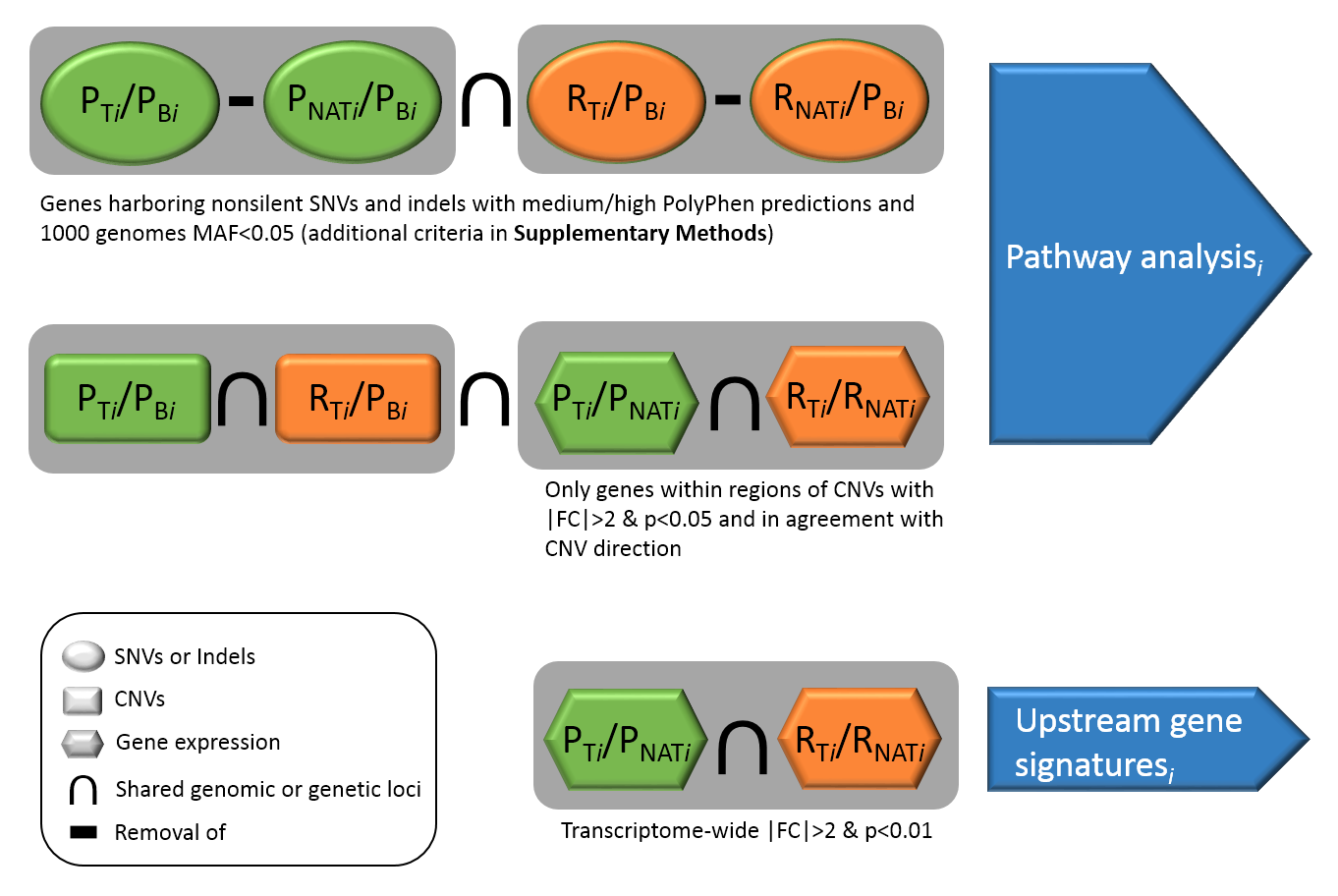


**Supplementary Figure 7**: Integrated genomics and genetics analytical flow to determine pathways most altered for patient *i* for *i*=1,..4. Individual patient’s pathway analysis and upstream gene signatures were combined to provide the top altered pathway shared between primary and recurrent tumors, which was Wnt signaling. The same strategy was also implemented using just those genomics and genetics loci unique to the recurrent tumors, which identified cell cycle signaling as the most activated. P_T_=primary tumor; P_NAT_=normal tissue adjacent to primary tumor; P_B_=recipient blood; R_T_=recurrent tumor; R_NAT_=normal tissue adjacent to recurrent tumor. Additional detail on methodology is described in the **Patients and Methods**. FC=fold change; MAF=minor allele frequency; SNVs=single nucleotide variants; indels=insertions or deletions.


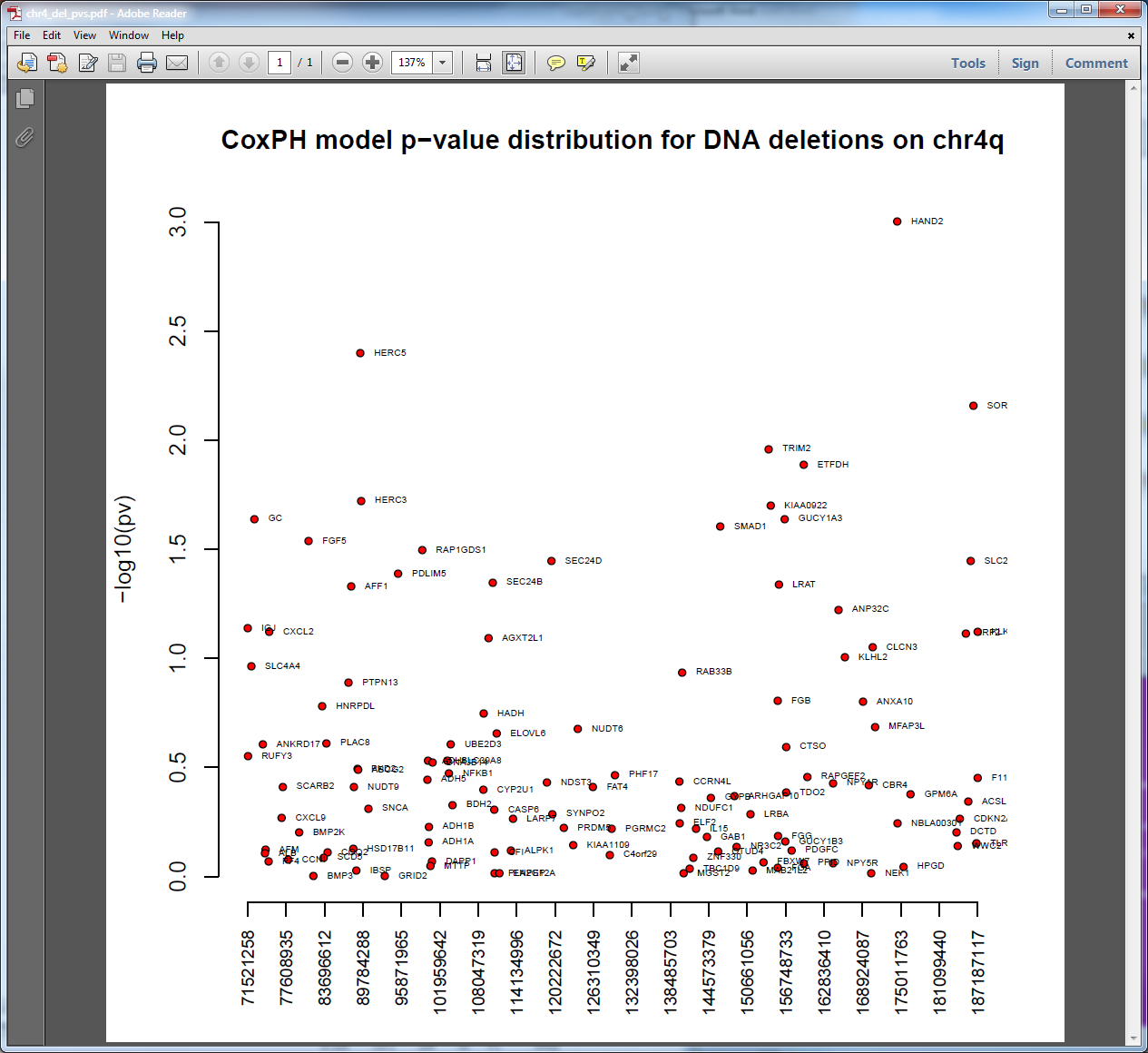


**Supplementary Figure 8:** Distribution of transformed p-values for *HERC5* high/low group from Cox PH regression model predicting HCC recurrence for genes within chromosome 4q deletion (x-axis is physical coordinates) using the Roessler et al. study.


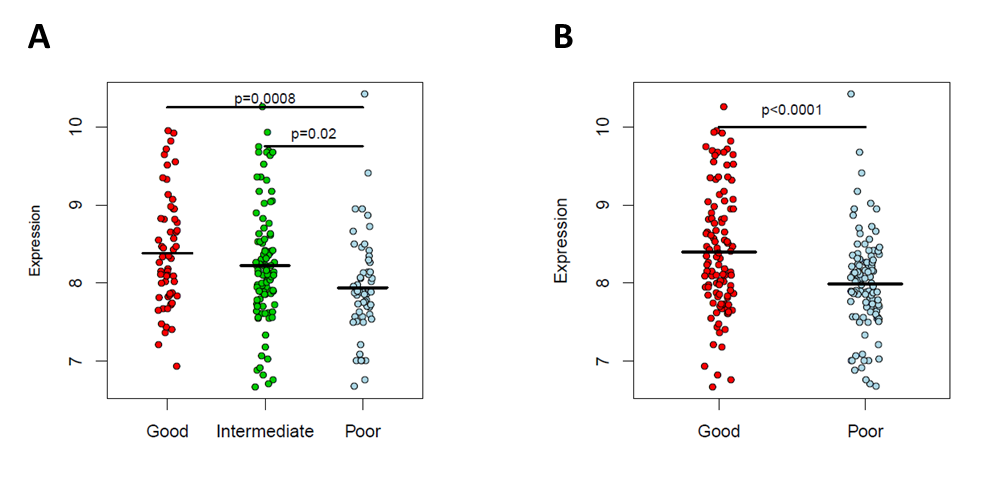


|  | poor | intermediate | good |
| --- | --- | --- | --- |
| Death rates | 4.60% | 1.50% | 1.10% |
| 10-year survival rates | 63% | 74% | 85% |
| 10-year HCC development rates | 42% | 28% | 18% |
| Annual HCC development rates | 5.80% | 2.20% | 1.50% |

**Supplementary Figure 9:** Expression profile of *HERC5* correlates with 186-gene signature used to predict prognosis (good=red; green=intermediate; light blue=poor) of patients with HCV-positive cirrhosis in liver specimens. **A)** Distribution of 3 prognoses levels determined from Hoshida et al, study (good: n=60; intermediate: n=101; poor: n=55). **B)** Distribution of 2 prognoses levels determined from Hoshida et al, study (good: n=109; poor: n=107). Table indicates risks calculated from time-to-event analyses using the 186-gene signature. Data taken from Hoshida et al, 2013.


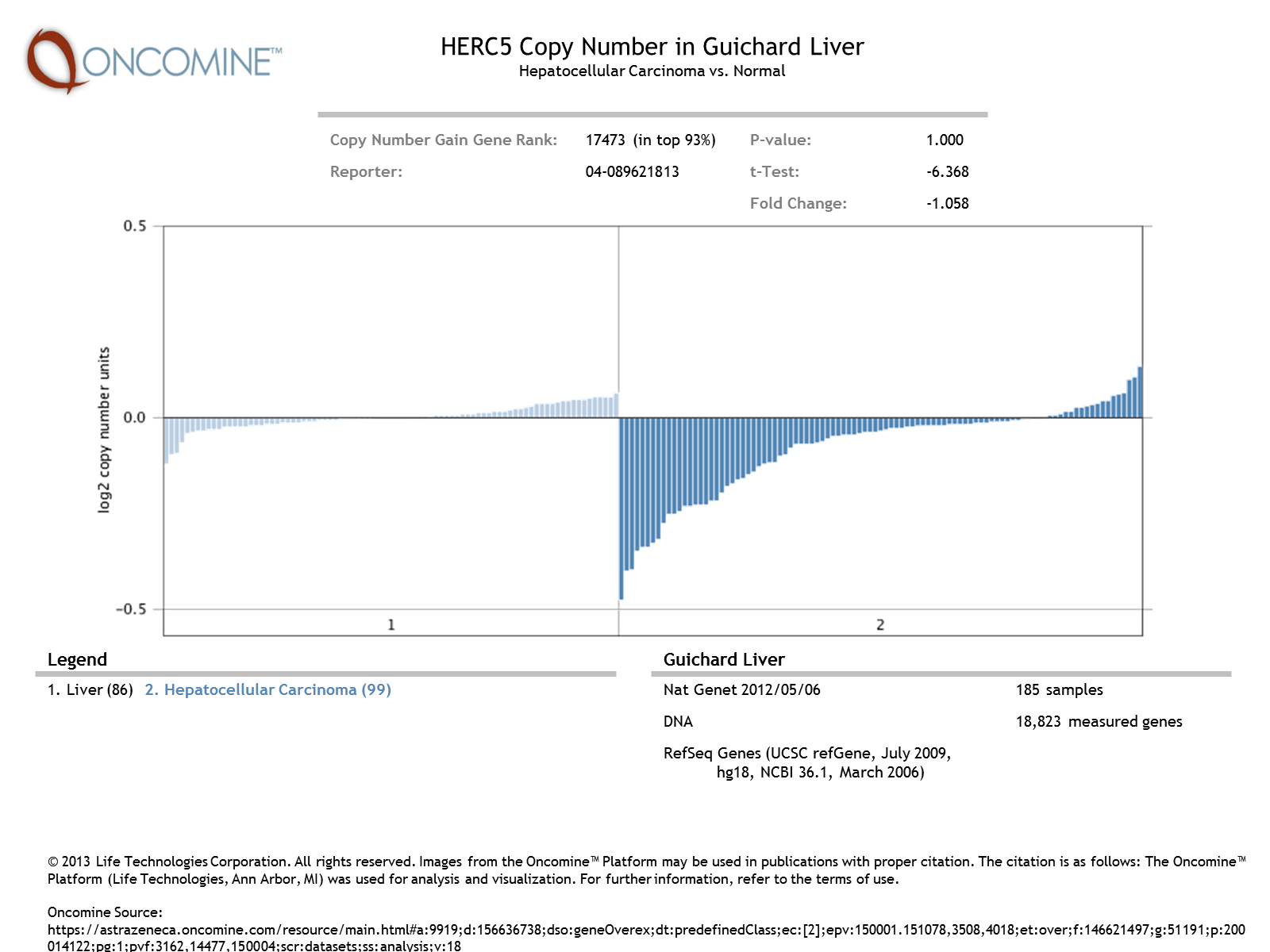


**Supplementary Figure 10:** Screenshot of the HCC patient distribution of HERC5 deletion from Guichard et al, from Oncomine (Life Technologies). A total of 28% of HCC patient primary tumors have a log ratio less than the minimum log ratio in the normal liver tissue distribution. A total of 78% primary tumors from HCC patients have a value 2-fold less than the median of the normal liver tissue distribution.
